# The Underlying Molecular Mechanisms Involved in Traditional Chinese Medicine *Smilax china* L. for the Treatment of Pelvic Inflammatory Disease

**DOI:** 10.1155/2021/5552532

**Published:** 2021-04-08

**Authors:** Yunsen Zhang, Zikuang Zhao, Huimin Chen, Yutong Fu, Wenxiang Wang, Qi Li, Xuanhao Li, Xiaobo Wang, Gang Fan, Yi Zhang

**Affiliations:** ^1^School of Ethnic Medicine, Chengdu University of Traditional Chinese Medicine, Chengdu, China; ^2^West China School of Medicine, Sichuan University, Chengdu, China; ^3^School of Pharmacy, Chengdu University of Traditional Chinese Medicine, Chengdu, China; ^4^Innovative Institute of Chinese Medicine and Pharmacy, Chengdu University of Traditional Chinese Medicine, Chengdu, China; ^5^Ethnic Medicine Academic Heritage Innovation Research Center, Chengdu University of Traditional Chinese Medicine, Chengdu, China; ^6^NMPA Key Laboratory for Quality Evaluation of Traditional Chinese Medicine (Traditional Chinese Patent Medicine), Chengdu University of Traditional Chinese Medicine, Chengdu, China

## Abstract

*Smilax china* L. (SCL) is extensively used in the treatment of pelvic inflammatory disease (PID). This study aimed to clarify the potential active ingredients of SCL and mechanisms on PID. SCL was widely distributed in Japan, South Korea, and China, which was traditionally considered heat-clearing, detoxicating, and dampness-eliminating medicine. Systems pharmacology revealed that 32 compounds in SCL may interact with 19 targets for immunoenhancement, antiapoptosis, anti-inflammation, and antioxidant activity of the PID model. Molecular docking revealed that isorhamnetin, moracin M, rutin, and oxyresveratrol may have higher binding potential with prostaglandin-endoperoxide synthase 2 (PTGS2), mitogen-activated protein kinase 1 (MAPK1), siderocalin (LCN2), tumor necrosis factor (TNF), and matrix metalloprotein-9 (MMP9), respectively. Molecular dynamics simulation showed that the binding modes of moracin M-MAPK1, rutin-TNF, and oxyresveratrol-MMP9 complexes were more stable, evidenced by relatively smaller fluctuations in root mean square deviation values. Conclusively, SCL may treat PID by inhibiting inflammatory factors, antitissue fibrosis, and microbial growth.

## 1. Introduction

Pelvic inflammatory disease (PID), the infection and inflammation of the female upper genital tract, is a common cause of infertility, chronic pain, and ectopic pregnancy [1]. Diagnosis and management are challenging, largely due to a polymicrobial etiology that is not fully delineated [2]. Reportedly, it is estimated that 2.5 million American women aged between 18 and 44 have received a PID diagnosis in their lifetime [3], and one in eight women with a history of PID encountered difficulties in getting pregnant [4]. PID treatment is mainly based on broad-spectrum antibiotic regimens and surgical treatment [2,5]. Antibiotics are effective in lessening short-term morbidity but have no effects on long-term complications, due to the disease's complex mechanism and long-term process [6]. Although the incidence of PID has decreased because of screening for gonorrhea and chlamydia and the early intervention of broad-spectrum antibiotics, damage to the reproductive system caused by infection has not been ameliorated [7]. Therefore, the therapeutic goal for the treatment of PID ought to include both short-term microbiological effects and long-term prevention of sequelae [6]. Also, the use of antibiotics is limited by the emergence of antibiotic resistance and PID without an identified pathogen. To inhibit progress, alleviate the long-term sequelae of PID, and avoid antibiotic resistance, it is often used in conjunction with traditional Chinese medicine (TCM) [8, 9]. Recent studies showed that the anti-inflammatory and immune mechanisms of PID were related to T cells, B cells, IG, cytokines (e.g., IL-6, TNF-*α*, and IL-1*β*), prostaglandin-endoperoxide synthase 2 (PTGS2), matrix metalloprotein-9 (MMP9), and TLRs signaling pathways [8, 10].

**Figure 1:**
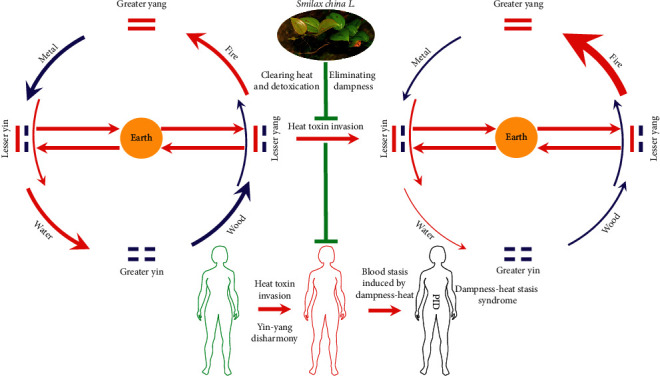
Effects of SCL on PID from view point of traditional Chinese medicine. From the theoretical perspective of TCM, the left and right circles represent the development process from physiological state of human (arrows of five phases are of the same size) to pathological state of PID patients (fire phase increased and water phase decreased). And the main interfering process of SCL against PID is between two circles. Above all, the internal pathogenesis of PID is the disharmony of yin-yang (a general term for all kinds of pathological changes due to imbalance and incoordination of yin and yang) and insufficiency of healthy qi (a collective designation for all normal functions of the human body and the abilities to maintain health, including the abilities of self-regulation and adaptation), and the external is that the dampness-heat and heat toxin invaded the thoroughfare and conception vessels, uterus, and uterine vessels. It caused blood blockage and abdominal pain. Hence, the core excitation of PID onset is the blood stasis induced by dampness-heat.

**Figure 2:**
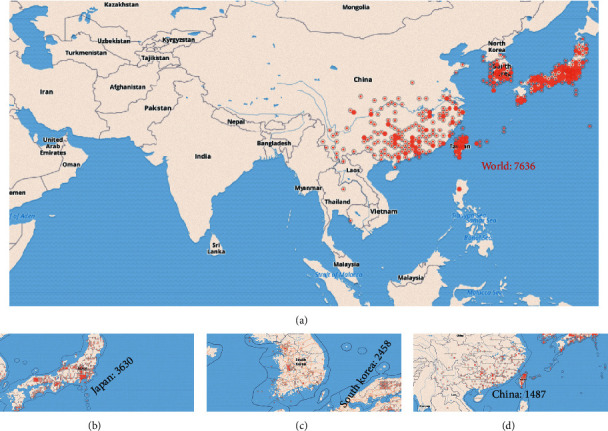
Distribution map on *Smilax china* L. by GBIF Secretariat online database.

**Figure 3:**
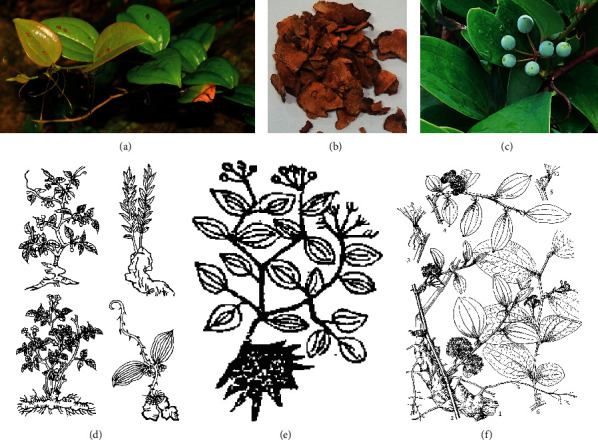
Original botany map and illustration of SCL plants in materia medicas. (a) The form of lamina and stem in SCL; (b) The Rhizoma pieces; (c) fruits. Its diversified styles of freehand sketching were recorded in *Chongxiu Zhenghe Jingshi Zhenglei Beiyong Bencao* in 1249 AD (d), *Compendium of Materia Medica* in 1552 AD (e), and *Flora of China* in 2004 AD (f).

From the theoretical perspective of TCM, the internal pathogenesis of PID is the disharmony of yin-yang (a general term for all kinds of pathological changes due to imbalance and incoordination of yin and yang) and insufficiency of healthy qi, and the external is that the dampness-heat and heat toxin invaded the thoroughfare and conception vessels, uterus, and uterine vessels. It caused blood blockage and abdominal pain (Figure 1). Hence, the core excitation of PID onset is the blood stasis induced by dampness-heat (dampness-heat syndrome) [11, 12]. *Smilax china* L. (SCL), a Liliaceae plant, commonly known as “Baqia” (or “Jin Gang Teng”), is widely distributed in Asia (Figure 2). From 1887 to 2020, it was found 7636 times (Figure 2(a)) according to GBIF online database (https://www.gbif.org/species/5295472/metrics) and mainly distributed in Japan (3630 times) (Figure 2(b)), South Korea (2458 times) (Figure 2(c)), and China (1487 times) (Figure 2(d)). Its original plant is shown in Figure 3, including the form of lamina and stem (Figure 3(a)), Rhizoma pieces (Figure 3(b)), and fruits (Figure 3(c)). Its diversified styles of freehand sketching were recorded in *Chongxiu Zhenghe Jingshi Zhenglei Beiyong Bencao* in 1249 AD (Figure 3(d)), *Compendium of Materia Medica* in 1552 AD (Figure 3(e)), and *Flora of China* in 2004 AD (Figure 3(f)). Up to now, SCL has been included in Chinese pharmacopeia [13] with the effects of heat-clearing, detoxicating, and dampness-eliminating. It has still been widely used in TCM for the treatment of PID and formulated into granules, syrup, pills, and capsules, demonstrating a good curative effect [9, 14]. Based on the previous studies, steroid saponin, flavonoids, glycosides, and stilbenes are the principal chemical compounds in SCL and demonstrated anti-inflammatory effect via TLR-4-mediated signaling pathway [14–16]. The flavonoid derivatives such as engeletin, isorhamnetin, and quercetin are the main constituents for the treatment of PID by inhibiting extracellular regulatory protein kinase and SMAD2/3 protein phosphorylation, thereby relieving the degree of fibrosis in the uterus via ERK1/2 and TGF*β*-SMAD2/3 signaling pathways [17]. However, most of these studies showed the rough mechanisms of extract in SCL against PID, and the main active compounds and how these compounds interact with PID-related targets to interfere with relevant signaling pathways are still unclear.

**Figure 4:**
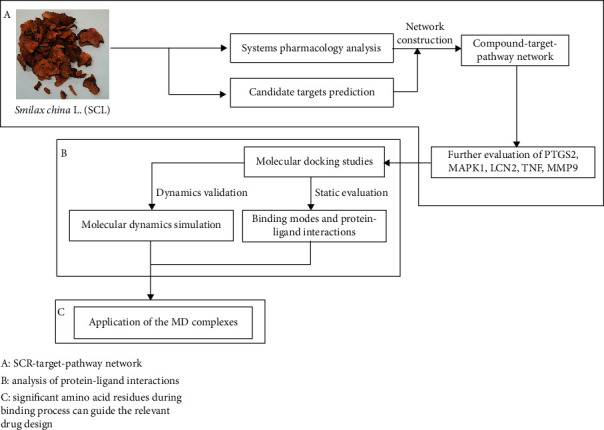
A schematic representation of workflow in this study.

TCM is designed to maintain the balance of the body's functions utilizing a lot of intricate compounds in herbs. Because multiple constituents may produce synergistic regulation on different targets, elucidating the mechanisms of TCM always takes lots of time and resources. There is no doubt that systems pharmacology has recently emerged as a new field including physiology, genetics, biochemistry, and molecular simulation via integrating various research methods to investigate the complicated mechanisms of multiple compounds [18, 19]. Nowadays, systems pharmacology has been applied for revealing the pharmacological mechanism of TCM from the perspective of entirety. For instance, most of the ingredients from well-researched herbs are carried out by molecular simulation, such as pharmacophore matching and inverse-docking to clarify the candidate targets which is available for researchers to further illustrate the integral mechanism of TCM [20–22]. Otherwise, the particle interaction, a role of the fundament of integral regulation, is the same concerned. Thus, static molecular docking and calculation of molecular mechanics-generalized Born surface area (MM-GBSA) free binding energy will provide a view of good binding pose and binding free energy to ensure that the complexes of compounds and targets possess enough energy to engender reaction of biochemistry [23, 24]. Finally, molecular dynamics (MD) simulation is utilized to evaluate the stability of protein-ligand complexes obtained from molecular docking using root mean square deviation (RMSD) and explore the noncovalent interaction between the active ingredients of herbs and the predicted targets, such as hydrogen bond (H-bond) and decomposition of molecular mechanics-Poisson Boltzmann surface area (MM-PBSA) energy of amino acid residues [25]. Overall, a schematic representation of the workflow in this study is shown in Figure 4, which makes it possible to systematically decode the active compounds and mechanism of TCM in the network. Hence, we evaluated the whole candidate targets of active compounds and provided a perspective of the integral mechanism via enriching the functions of targets and dissected the molecular mechanism of SCL in the treatment of PID using computational systems pharmacology.

## 2. Methods

### 2.1. Screening of Potentially Active Compounds in SCL

SCL compounds were systematically listed as ligands from published paper mining [26, 27], TCMID (http://www.megabionet.org/tcmid/) [28], SymMap (https://www.symmap.org/detail/SMHB00008) [29], and TCMSP databases (http://tcmspw.com/tcmsp.php) [30]. All compound structures from PubChem (https://pubchem.ncbi.nlm.nih.gov/) [31] were filtered by utilizing the “Lipinski rules” of the Molinspiration database (https://www.molinspiration.com/cgi-bin/properties) [32]. In the field of drug discovery, the Lipinski rules were used to screen the compound database to eliminate molecules that were unsuitable for drug use, including n·OHNH ≤ 5, n·ON ≤ 10, MW ≤ 500, and miLogP ≤5. Compounds that met the Lipinski rules and others that did not but possessed good bioactivity were used in systems pharmacology and molecular docking [33, 34]. The 2D structures (.sdf format) of all compounds were generated by ChemBioOffice2014 [35].

### 2.2. Candidate Targets of Active Compounds in SCL

Compounds that satisfied Lipinski rules were uploaded to SwissTargetPrediction (http://www.swisstargetprediction.ch/) [36], PharmMapper (http://www.lilab-ecust.cn/pharmmapper/) [37], and SEA (http://sea.bkslab.org/) [38] to obtain preliminarily candidate targets. All the genes should be from “*Homo sapiens*” to clarify the function of critical targets and were proofread by the UniProt database (https://www.uniprot.org/) [39].

### 2.3. Network Construction of Compound-Target-Pathway in SCL for PID Treatment

Systems pharmacology was applied to analyze the interaction between SCL and PID and the selection of critical targets. To identify the intersection of targets between PID and SCL, the title of “pelvic inflammatory disease” was placed in GeneCards (https://www.genecards.org/) [40], DisGeNET (https://www.disgenet.org/) [41], DrugBank (https://www.drugbank.ca/) [42], and existing research [43,44] to obtain gene names of PID targets, which manually confirmed that each target had a clinical study in PID. Additionally, the intersection genes (compound- and disease-related targets) were used to perform annotation analysis of the obtained crossover genes by using Gene Ontology (GO) and the KEGG pathway analysis functions in the STRING platform (https://string-db.org/) [45], with the intersection genes directly mapped to the pathway. Cytoscape [46] was used to visualize a network of “Compound-target-pathway (C-T-P network)”. In this network, each compound, target, and pathway was indicated by nodes, and the interactions between each node were described by edges. The network was established to project an overview of the interactions among compounds, targets, and pathways.

### 2.4. Protein-Protein Interaction (PPI) Analysis of Crucial Targets in SCL for PID Treatment

To reveal the direct and indirect roles active compounds of SCL played in the pelvic inflammatory targets, the intersection targets were introduced to the STRING platform and a graphical network of PPI was generated [47]. In the network, each node represents all the proteins produced by a single, protein-coding gene locus, and edges represent protein-protein associations which are meant to be specific and meaningful, i.e., proteins jointly contribute to a shared function; this does not necessarily mean they are physically binding each other. Ultimately, to clarify the interfering mechanism, compounds broke into PPI network targets with a high degree and relating to the critical pathway in the PPI network were selected to operate molecular docking.

### 2.5. Docking between PID Crucial Targets and Active Compounds in SCL

Due to the algorithm defect of the database on target prediction, a thorough docking was performed to improve the credibility of systems pharmacology. The crystal structures (.PDB) of selected targets were obtained from RCSB (http://www.rcsb.org/) [48], and the crucial targets were docked with active compounds.

Schrödinger Glide was used to pretreat the 3D protein structure for docking, including adjusting the bond orders to ensure the stability of the chemical bonds between the atoms, adding the missing hydrogen atoms and amino acid residues, optimizing the orientation of amino acids and hydrogen atoms, optimizing the distribution of H-bonds, and removing water molecules and heterogeneous molecules. Finally, energy minimization with a force field OPLS-2005 was supplied.

At the end of pretreatment, the Receptor Grid Generation module was employed to select the ligand-binding cavity and generate a grid in protein. Next, the active compounds were introduced to Maestro and optimized by the liquid simulation of OPLS-2005 all-atoms force field in the LigPrep module, as well as combined into a ligand package. The docking accuracy was evaluated by standard precision (SP), as well as flexible docking. At this point, the docking preparation was completed, the scaling factor and partial charge cutoff of van der Waals radius scaling 1.0 and 0.25 were used to generate the grids on active sites, and ligand package was selected to perform molecular docking in the Ligand Docking module. Next, the generated Glide G-score was used to assess the affinity between compounds and proteins. Moreover, crucial targets were docked with their self-ligands to set positive contrast, and their Glide G-scores were used to measure whether compounds possessed a good affinity to the protein and standardize the score of compounds to be visualized as a heat map by MeV [49] (step 1: normalized genes/rows; step 2: hierarchical clustering: average dot product-complete linkage clustering).

### 2.6. Binding Free Energy Calculation (MM-GBSA) Based on SP Docking

Good poses and good scores were obvious by SP docking, but what was the binding free energy of the docked complex was another problem. Docking results showed the active compounds did bind to the active site of the protein, but could this association last long enough to elicit any potential biological response, as biological response largely depends upon the binding free energy of the association. Therefore, the docked complexes in SP mode were subjected to binding free energy calculation (MM-GBSA) using the Prime module of Maestro [50]. A total of 14 active compounds were selected for this analysis.

### 2.7. Molecular Dynamics Simulation

MD simulation is a powerful method in the analysis of the target-ligand interactions by considering the flexibility of binding pose. A good pose of complexes, an essential premise of MD, is obtained by molecular docking. Otherwise, all atoms of the system in complexes are sanctioned to motion and interact for a fixed period (∼45 ns), and the trajectories of atoms and molecules are defined through Newton's equations of motion. In this study, the best poses of SP docked ligand/protein with an excellent binding free energy were employed to operate explicit solvent MD simulation. GROMACS [51] as a computational tool of MD simulation was used for this purpose.

Due to five complexes that would perform MD simulation, we had to protocol slightly various systems to reach our expectation as shown in Supplementary Table 1. In general, coordinates and charge of ligands were generated using PRODRG 2.5 [52], and protein and SPC water model were described by the GROMOS96 43A1 force field [53] and defined in a solvent box with 8.5 nm × 8.5 nm × 8.5 nm. Na^+^ and Cl^–^ions were added to ensure the overall neutrality of the systems. Each MD simulation system was first relaxed to remove possible steric crashes by the steepest descent energy minimization algorithm and stopped minimization when the maximum force is <100.0 KJ/mol. In the second step, a 100 ps simulation was performed utilizing the canonical ensemble (NVT ensemble) using the modified Berendsen thermostat with a slowly ascending temperature from 0 K to 300 K, a fast temperature relaxation constant of 0.1 ps, and a temperature coupling of protein and ligand to prevent system bursting. Next, the periodic boundary condition was employed to produce the constant temperature and pressure (NPT) ensembles. The pressure was set at 1.0 bar and was controlled by the isotropic pressure scaling protocol applied in GROMACS. Moreover, no cutoff limit was used for electrostatic forces by employing the particle mesh Ewald (PME) algorithm. All bonds were constrained using the LINCS algorithm. Then the simulation time for each system was 45 ns, and the trajectories of simulated systems were saved every 10 ps. Finally, disintegrated calculation of binding free energy was hired to qualitatively analyze the principal force of interaction between protein and ligand. All results were visualized by QtGrace (https://sourceforge.net/projects/qtgrace/), VMD (http://www.ks.uiuc.edu/Research/vmd/), and LigPlot^+^ (https://www.ebi.ac.uk/thornton-srv/software/LigPlus/).

## 3. Results

### 3.1. Compound-Target-Pathway Network of SCL in the Treatment of PID

**Figure 5:**
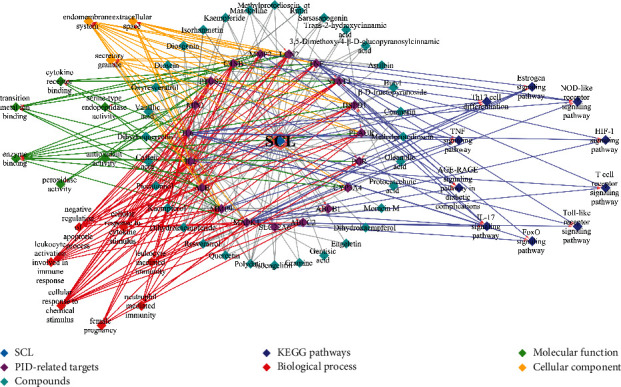
Compound-target-pathway (C-T-P) network. The network consists of compounds, targets, and pathways, including 78 nodes and 285 edges. 32 components interact with 19 target proteins and are associated with PID through 10 pathways and 16 GO annotations.

After filtering was operated for Lipinski rules, thirty-two in sixty-eight compounds were screened as active compounds in SCL and are listed in Supplementary Table 2. Chemical formats of smile and SDF were generated to predict the potential targets. Then 718 potential targets (some were duplicates) associated with compounds were screened from the SwissTargetPrediction, PharmMapper, and SEA server, respectively (Supplementary Table 3). Otherwise, eighty-six PID-related targets were retrieved from databases (Supplementary Table 4). Then, to clarify the relationship between herb and disease, a network of compounds, targets, and pathways was visualized by Cytoscape. We found that a total of 32 compounds could act on 19 key targets and associate with 16 relevant GO annotations and 10 effective pathways (Figure 5).

### 3.2. PPI Network Analysis

**Figure 6:**
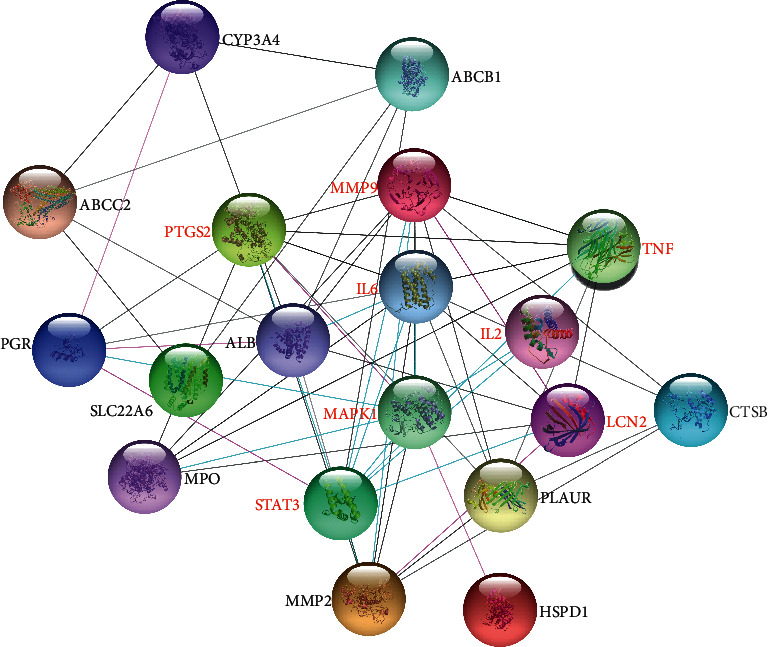
Protein-protein interaction analysis of 19 PID-related targets. Red labels were used to highlight the IL-17 signaling pathway (MMP9, TNF, IL6, PTGS2, LCN2, and MAPK1) and Th17 cell differentiation (IL2, MAPK1, IL6, and STAT3).

19 intersection targets were analyzed using the PPI network in the STRING platform (Figure 6). Relevant parameters of the network were as follows: (1) number of nodes and edges: 19 and 91; (2) average node degree and expected number of edges: 9.58 and 26; and (3) PPI enrichment *p* value: <1.0*e* − 16. Otherwise, red nodes (MMP9, tumor necrosis factor (TNF), interleukin-6 (IL-6), PTGS2, neutrophil gelatinase-associated lipocalin (LCN2), mitogen-activated protein kinase 1 (MAPK1), interleukin-2 (IL-2), and signal transducer and activator of transcription 3 (STAT3)) were used to highlight the IL-17 signaling pathway and Th17 cell differentiation. All highlighted targets except IL-2, STAT3, and IL-6 were selected as core targets to illuminate the SCL interfering mechanism against PID utilizing molecular docking and MD simulation.

### 3.3. Molecular Docking with Binding Free Energy

**Figure 7:**
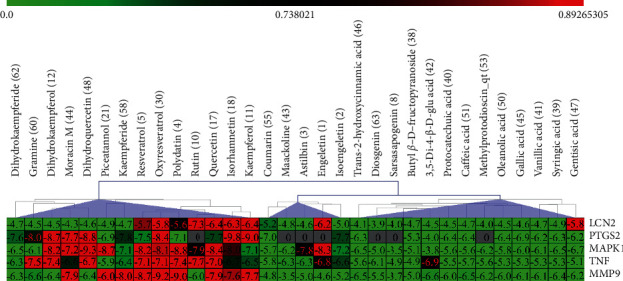
Clustering heat map between compounds and PID-related targets. All compounds with a docking score of proteins were divided into 3 clusters from left to right considering the affinity. 14 active compounds on the right were operated MM-GBSA binding free energy. “Di”: dimethoxy; “glu”: glucopyranosyl cinnamic.

The heat map was employed to stick out the features of 32 active compounds as shown in Figure 7 (original data are presented in Supplementary Table 5). Compounds that had a high activity clustered together excellently, with a high affinity to PTGS2, LCN2, TNF, MAPK1, and MMP9. Rutin (10), isorhamnetin (18), oxyresveratrol (30), and moracin M (44) were found to occupy the top score, which exceeded or neared the original ligands in verified docking. Furthermore, compounds demonstrated a binding affinity to one or several targets. According to the distance metric of average dot product in Mev, 14 capital protein-ligand molecular interactions were analyzed in Table 1.

**Figure 8:**
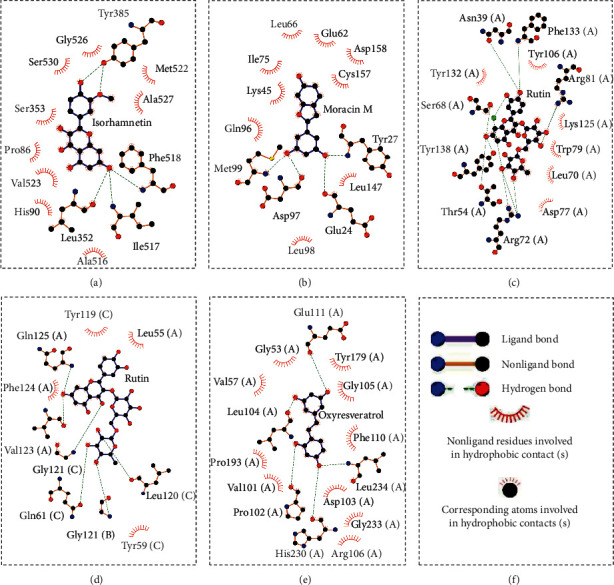
Docking poses between compounds and PID-related targets. (a) Isorhamnetin and PTGS2; (b) Moracin M and MAPK1; (c) Rutin and LCN2; (d) Rutin and TNF; (e) Oxyresveratrol and MMP9. (a–e) 2D binding models within 4 Å residues.

Further analysis (Figure 8) demonstrated that rutin possessed a strong binding ability to the carboxymycobactin binding cavity [54] of LCN2 and inhibitor binding cavity [55] of TNF (Figure 8(c): rutin and LCN2; Figure 8(d): rutin and TNF). Otherwise, as shown in Figure 8(a), isorhamnetin (–51.06 kcal/mol) formed four H-bonds with GLN 192, PHE 518, TYR 385, and SER 530 indicating that was matched well in the rofecoxib (a COX-2 inhibitor) binding pocket [56] of PTGS2.  Figure 8(b) demonstrates that moracin M (−37.01 kcal/mol) separately formed one interaction of pi-cation and three H-bonds with LYS A 45, GLU A 62, GLN A 96, and GLU B 360, affecting the biding cavity of allosteric and ATP-competitive inhibitor [57] of MAPK1. Finally, as for the inhibitor binding activity [58] of MMP9, Figure 8(e) indicated that oxyresveratrol formed two pi-pi stacking with TYR 179 and PHE 192 and three H-bonds with ALA 191, HIS 210, and GLY 233, which illustrated the formation process of a good pose.

### 3.4. Molecular Dynamics Simulation of Five Docking Poses

#### 3.4.1. Root Mean Square Deviation and Total H-Bonding Change Analysis

**Figure 9:**
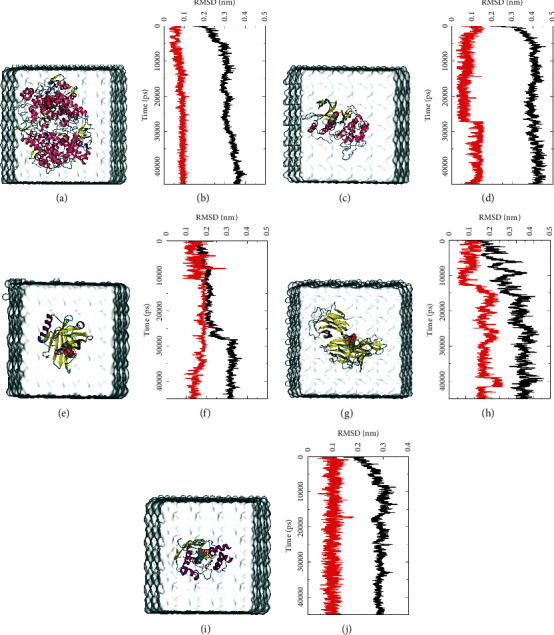
The 45 ns molecular dynamics simulation and calculated root mean square deviations (RMSD) of the backbone atoms and ligands referenced to the topology documents of five complexes. (a, b): PTGS2-isorhamnetin; (c, d): MAPK1-moracin M; (e, f): LCN2-rutin; (g, h): TNF-rutin; (i, j): MMP9-oxyresveratrol. (a, c, e, g, i) Simulation system for the protein-ligand model complex, with water in cyan box, protein in new cartoon of secondary structure, and ligand in VDW representation. (b, d, f, h, j) RMSD fluctuation for proteins and ligands.

RMSD is of importance to quantify the structural stability of protein-ligand complexes within a fixed time frame [59]. In this study, five complexes were used to calculate the RMSD within 45 ns. Firstly, RMSD analysis and complexes in solvent depicted that PTGS2 (Figures 9(a) and 9(b)) started to stabilize after 10 ns, and it maintained 20 ns stability until 30 ns and slightly increased after 30 ns. Interestingly, a similar fluctuation was captured in the change of total H-bonds at 30 ns (Supplementary Figure 1A), and the average number of H-bonds before 25 ns was 2.7 but increased to 3.5 during the last 15 ns. This fluctuation indicated that the stable growth of the H-bond and the transition of one stabilized configure to another. The ligand (Figure 9(b)) started stabilizing after 10 ns and maintained to 45 ns. The average RMSD of protein and ligand was 0.312 and 0.082 nm, respectively.

Secondly, MAPK1-moracin M complex (Figures 9(c) and 9(d)) gained stability at around 10 ns, and the RMSD pattern of ligand suddenly increased at 27 ns and maintained to 45 ns. At the same time, the corresponding change appeared in the number of total H-bonds (Supplementary Figure 1B). The average number of H-bonds before 27 ns was 3.7 and increased to 4.1 during the last 18 ns. The average RMSD of protein and ligand was 0.286 and 0.116 nm, respectively.

Thirdly, for the LCN2-rutin system (Figures 9(e) and 9(f)), stable protein-ligand interaction was also observed. The protein and rutin were both equilibrated at ∼11 ns with a slight fluctuation. The main fluctuation of protein was observed at 27∼30 ns and equilibrated during the last 15 ns. In the view of total H-bond number (Supplementary Figure 1C), the H-bonds of complex initially increased before ∼22 ns (average number: 2.8), reduced by 0.7 at 22∼32 ns, and stabilized at 3.2 during the last 13 ns. The average RMSD of LCN2 and rutin was 0.247 and 0.164 nm, respectively.

Fourthly, for multiple chain (four chains) system of TNF-rutin (Figures 9(g) and 9(h)), it was time-consuming to equilibrate. Therefore, a stable protein-ligand interaction was observed during the last 15 ns. The RMSD of TNF constantly rose before ∼26 ns. The ligand initially stabilized at ∼16 ns with a slight fluctuation and ultimately stabilized at 30 ns, and a sudden increase in the RMSD of ligand lasted 3 ns was captured. The total H-bond number was selected for analysis during the last 20 ns. A constant change and a large span of the H-bond number were recorded before 30 ns, which indicated an unstable H-bonding. Then, the span of H-bond variation gradually reduced, and 2∼4 stable H-bonds were retained to the end. The average RMSD of TNF and rutin was 0.389 and 0.189 nm, respectively.

Finally, for the MMP9-oxyresveratrol system (Figures 9(i) and 9(j)), the RMSD analysis depicted that the protein achieved stability at around 7 ns and fluctuated slightly at ∼16 ns, and the ligand equilibrated at 1 ns and maintained until 40 ns with a slight fluctuation at ∼ 17 ns. Meanwhile, the change of the H-bond number gradually stabilized in the last 25 ns. The average RMSD of MMP9 and oxyresveratrol was 0.282 and 0.097 nm, respectively. Overall, the RMSD and total H-bond change analysis of five MD simulation complexes illustrated that the all above systems maintained a period of stability with ligands.

#### 3.4.2. Intermolecular H-Bonding

Hydrogen bonding is among the most essential parameters to understand the binding affinity of small molecules towards a biomacromolecule (e.g., protein). A large number of H-bonds present in between protein and small molecules signify a strong binding affinity. In this regard, H-bonds between the natural molecules and disease-related proteins were monitored over the MD simulation time.

The H-bond analysis results are shown in Tables 2 and 3. In the PTGS2-isorhamnetin complex, a total of 18 different H-bonds (H-bonds between ligands and different residues in all frames) were detected. On average, there were 2.5 H-bonds (average number of hydrogen bonds as a function of time), and the distance and angle were 2.94 Å and 14.88°, respectively. The amino acid residues on the side chain including H-bond donor (Phe 518, Ile 517, and Ser 530) possessed high occupancy to isorhamnetin.

In the case of MAPK1, a total of 19 different H-bonds were detected. On average, there were 4.0 H-bonds, and the distance and angle were 2.85 Å and 16.15°, respectively. The H-bond occupancy of important amino acid residues on the main chain and side chain including acceptor (Glu 62 and Asp 97) and donor (Tyr 27 and Met 99) was all greater than 60%, and the occupancy of Glu 62 with moracin M was 124.50% which showed that one more H-bond existed. And this result was in agreement with molecular docking.

In the case of LCN2, a total of 45 different H-bonds were detected. On average, we observed 2.8 H-bonds, and the distance and angle were 2.85 Å and 14.59°, respectively. The amino acid residues on the side chain including H-bond donor (Ser 68 and Arg 81) and acceptor (Tyr 106 and Tyr 138) possessed good occupancy and were also in agreement with molecular docking.

In the TNF-rutin complex, 25 different H-bonds were detected. On average, we observed 2.2 H-bonds, and the distance and angle were 2.94 Å and 16.93°, respectively. Due to a constant change and large span of the H-bond number mentioned in “Section 3.4.1”, the occupancy of amino acid residues including H-bond receptor (Val 123 main and Tyr 119 side) and donor (Ser 60 main) was generally lower.

As for the MMP9-oxyresveratrol complex, 20 different H-bonds were detected. There were on average 4.3 H-bonds, and the relevant distance and angle were 2.78 Å and 14.78°, respectively. Three H-bond receptors (e.g., Leu 104 main, His 230 main, and Pro 102 main) and one donor (Leu 234 main) were bound to oxyresveratrol stably. These results hinted that the natural molecules interacted effectively towards the active site of PID-related proteins with a significant property of H-bonds.

#### 3.4.3. Energy Decomposition of MM-PBSA

**Figure 10:**
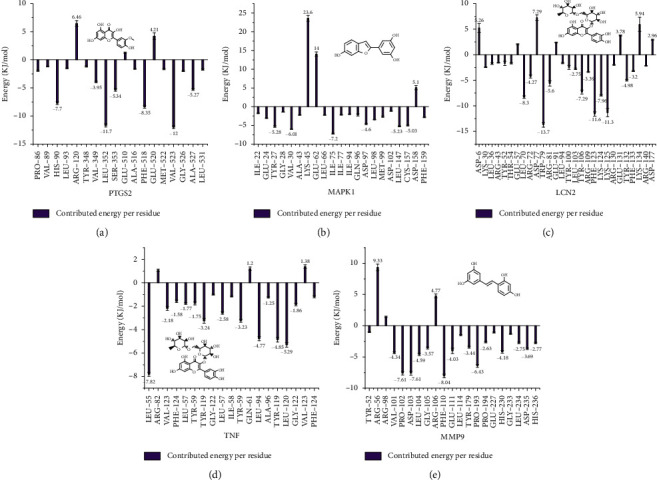
The energy contribution of important amino acid residues of five protein-ligand complexes. The significant residues of energy contribution (e.g., Val 523 and Leu 352 on PTGS2, Ile 75 on MAPK1, Trp 79 on LCN2, Leu 55 on TNF, and Phe 110 on MMP9). (a–e) The positive residues and negative residues (only residues which were out of the range: -1∼1 KJ/mol remained). Most of the residues within 4 Å play positive roles for lower energy saving Lys 45 & Glu 62 on MAPK1 and Arg 56 & Arg 106 on MMP9.

To explore the interaction between proteins and their ligands, energy decomposition of each complex was performed by using g_mmpbsa [60] module of GROMACS software. A total of 100 snapshots were extracted from the stable and continuous trajectories for the free energy calculation. The binding free energy could be divided into van der Waals Interaction (∆Evdw), electrostatic energy (∆Eele), polar solvation interaction (∆Epol), and solvent-accessible surface area (∆Esasa). The results were listed in Table 4 and Figure 10. To understand the energy contribution, ∆Bind (−146.535 ± 1.934 KJ/mol) of the PTGS2-isorhamnetin complex was decomposed to each amino acid residue. Figure 10(a) hinted that the important residues including Val 523, Leu 352, Phe 518, and His 90 involved in the binding site of PTGS2 (Figure 8(a)) showed a positive tendency for binding. As Figure 10(b) shows, Lys 145, Glu 62, and Asp 158 in MAPK1 showed a negative tendency to bind, and the latter two residues were within 4 Å of ligand, but Glu 62 was the important residue which formed excellent H-bond interaction with the ligand. Otherwise, residues (e.g., Val 30 and Ile 75) that showed positive binding tendency were within 4 Å to the ligand. The results showed that residues which had a positive binding tendency were closer to ligands (within 4 Å) and even formed H-bonds and pi-pi stacking interaction, and the negative residues were apt to far away from the ligand as shown in Figures 10(c)–10(e). All the above results were consistent with our previous docking results.

## 4. Discussion

Traditionally, in modern medicine, drugs were designed to target specific proteins relevant to the disease. However, herbs could possess even hundreds of compounds with multiple targets in TCM, which presented a significant obstacle to the exploration of drug mechanisms [61]. Fortunately, systems pharmacology, molecular docking, and MD simulation provided a holistic perspective to clarify the potential mechanisms for illustrating the integral regulation-based active compounds of TCM and conducting the consequent experiment [19]. Therefore, the above methods were employed to reveal the intricate mechanisms of SCL in the treatment of PID.

**Figure 11:**
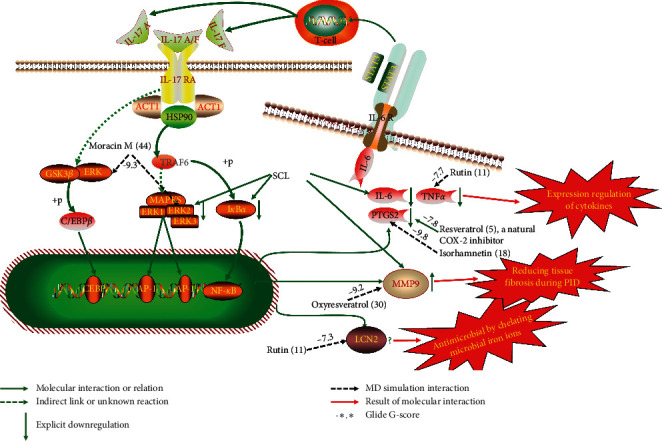
Computational simulation and experimentally validated regulation mechanism of SCL in PID model.

According to the C-T-P network (Figure 5), we proposed a simple inference as shown in Figure 11 (details are presented in Supplementary Figure 2), with the IL-17 signaling pathways (the highest value of −log10 (FDR)) considered potentially efficacious utilizing in-depth excavation of the above network. IL-17 signaling pathway reportedly had dual regulatory roles in proinflammatory and host defense processes [62, 63]. On the one hand, excessive secretion of IL-17A and IL-17F from Th17 cells can induce massive inflammatory factors including IL-6, IL-1*β*, and TNF-*α*. IL-17 signaling pathway can also synthesize prostaglandin E2 (PGE2) by inducing PTGS2, and the vasodilator effect of PGE2 also promoted inflammatory cells to enter the site of inflammation, so the activation of IL-17 signaling pathway had a strong proinflammatory effect [64]. In previous studies, SCL downregulated the expression of IL-6, interleukin-1 beta (IL-1*β*), TNF-*α*, IL-2, and PTGS2 in rats of the PID model, but its regulatory mechanisms involved remain unclear [17, 26]. However, combined with the results of this study, this may be the SCL inhibition of the IL-17 signaling pathway.

On the other hand, in the process of immunoregulation, the regulation of IL-17 signaling pathway can recruit neutrophil to the inflammatory region, releasing myeloperoxidase (MPO) [65] and inducing gene expression of LCN2 and matrix metalloproteinases (MMPs) [63, 66]. These proteins make important impacts in host defense, which may contribute to alleviating PID symptoms by enhancing the immune function of the body. For instance, MMP9 was activated to promote embryo formation, wound healing, and transfer of inflammatory cells [66–68]. Interestingly, it was reported that SCL upregulated the expression of MMP-2 and MMP-9 in rats of the PID model and downregulated the MMPs inhibitor TIMP-1, thereby restoring the balance between MMPs and TIMP-1 and reducing tissue fibrosis during PID [17]. Collectively, these results demonstrated the multitarget regulation of compounds in SCL.

Although the above network provided a clear view of the integral regulation of SCL, there was still a barrier to validate the facticity of each research data and it was difficult to consider the critical effect of each result. Hence, how to select a crucial result (targets or pathways) to concern and validate was an issue worth pondering carefully for researchers. Therefore, PPI analysis could play an essential role which helps us find the key targets and pathways and explore critical radioactive targets in the network. In this study, IL-17 signaling pathway and Th17 cell differentiation-related targets were considered focuses in the PPI. As previously reported, PTGS2 is inducible and usually produces inflammatory prostaglandins, which mediate responses to physiological stress (infection and inflammation), stimulate chronic inflammation, and is a target for nonsteroidal anti-inflammatory drugs (NSAIDs) [56]. As shown in Table 1 and Figure 8, the binding capacity of isorhamnetin to PTGS2 (Glide G-score: −9.757) was very close to rofecoxib (−9.800), suggesting that isorhamnetin may be a potential novel COX-2 inhibitor somewhat analogous to rofecoxib. Additionally, all the active compounds (Figure 7) mostly belonged to flavonoids and stilbenes. Based on existing pharmacodynamic investigations, these flavonoids and stilbenes have achieved obvious anti-inflammatory effects and ameliorated fibrosis in PID animal models by inhibiting the synthesis or release of histamine, 5-hydroxytryptamine (5-HT), and PGE2, as well as enhancing the production of MMP9 in uteri [17, 69]. Particularly, engeletin (1), polydatin (4), and resveratrol (5) have inhibited the release of IL-6 and TNF-*α* [70]. Rutin (10) inhibited the release of IL-2 and TNF-*α* [26]. These results and existing experiments indicated that active ingredients in SCL, including isorhamnetin (18), polydatin (4), oxyresveratrol (30), and piceatannol (21), could inhibit the activity of PTGS2 to decrease the synthesis of PGE2 [71], which contributes to the restoration of PID by inflammatory inhibition. What is more, MD simulation of five complexes during 45 ns was utilized to understand the dynamics binding process between proteins and ligands, which corresponded to molecular docking. Protein complexes (Supplementary Table 1) whatever single chain (LCN2 and MMP9) and multiple chains (PTGS2, MAPK1, and TNF) could stabilize ultimately (Figure 9). The fluctuation of various systems was significantly relevant to H-bonding change. As shown in Table 2, the properties of H-bond in different systems were decoded. For the MAPK1-moracin M and MMP9-oxyresveratrol systems, the average H-bond numbers were greater than the LCN2-rutin and TNF-rutin systems, but the detected H-bonds were lower than them. The structures of rutinose and flavonoid aglycones on rutin were easy to form H-binding interaction to surrounding amino acid residues in the protein. Otherwise, the H-bonding interaction and energy contribution of crucial residues were analyzed (Table 3 and Figure 10); the significant H-bond-related residues including Phe 518 on PTGS2, Glu 62 on MAPK1, Ser 68 on LCN2, Val 123 on TNF, and Leu 104 on MMP9 were of great importance to maintain the system's stability. The significant residues of energy contribution (e.g., Val 523 and Leu 352 on PTGS2, Ile 75 on MAPK1, Trp 79 on LCN2, Leu 55 on TNF, and Phe 110 on MMP9) indicated the van der Waals interaction and electrostatic energy were the same important. So far, a part of the results in network pharmacology has been validated and discussed via existing experiments, molecular docking, and MD simulation, which can yet be regarded as an effective method to elucidate the active compound of SCL and relevant mechanisms against PID. In our study, the protein LCN2 which can inhibit microorganisms by chelating the iron ions [72] was found to have an important impact and phenolic compounds (e.g., moracin M) were also considered as key compounds in treating PID. Mechanistically, compared to previous research studies on the main constituents for the treatment of PID by relieving the degree of fibrosis in the uterus via ERK1/2 and TGF*β*-SMAD2/3 signaling pathways, we reported the critical active compounds and relevant binding modes of IL-17 signaling pathway and Th17 cell differentiation-related targets in the treatment of PID by inhibiting inflammatory factors, antitissue fibrosis, and microbial growth.

However, some tough problems associated with disease targets and traditional herbs still bother investigators. The first thing is that the vague and a small number of valuable targets based on current research studies cannot orient to the whole disease. Therefore, systems pharmacology and molecular simulation are used to reveal the partly network mechanisms and molecular actions rather than the whole disease network mechanisms. And similar to the current drug discovery strategies, compared with the number of disease targets, the quality of the targets (druggability and crystal reliability) is paid more heed by researchers. Secondly, as for molecular docking, due to the limitation of computational power and force field algorithm, a newly and generally applicable force field to improve the accuracy of molecular docking and fast methods of the binding free energy calculation for virtual screening [73] are urgent to develop. Enough computational accuracy provides researchers enough confidence to conduct subsequent experiments. Finally, the specific components in TCM are needed to extract and identify completely to enrich the efficacious material basis. It is comparatively easy to illustrate the molecular mechanism once obtaining reliable targets and adequate ingredients. All above, there is a shortcut to uncover the overall network mechanism of TCM against diseases through cross cooperation among pharmacology, medicinal chemistry, and computational chemistry.

## 5. Conclusions and Further Prospect

Considering the promising treatment potential of SCL on PID, efforts are in demand to reveal the acting targets for SCL and the unclear mechanisms behind the therapeutic potentials. In this study, the computational systems pharmacology method was applied to explore the active ingredients of SCL and provided an integral view of the mechanism against PID. The principal 32 potent ingredients for the treatment of PID were uncovered to regulate 718 candidate targets. Furthermore, in the PPI and C-T-P network analysis, 8 of 19 PID-related targets were mapped to the IL-17-signaling pathway and Th17 cell differentiation. We focused on five reported PID-related targets PTGS2, MAPK1, LCN2, TNF, and MMP9. The interactions between active compounds and PID-related targets were described with static and dynamic evaluations. A total of 14 active compounds, including rutin (−40.46 kcal/mol), isorhamnetin (−51.06 kcal/mol), oxyresveratrol (−56.71 kcal/mol), and moracin M (−37.01 kcal/mol), showed greater binding force to the therapeutic targets. At the same time, the amino acid residues in the hydrophobic cavity which played an important role in the process of complexes were revealed to guide the design of relevant drugs.

Overall, active ingredients of SCL exhibited a strong affinity to therapeutic targets of PID, thereby contributing to decreasing inflammation, ameliorating fibrosis, and inhibiting or eliminating microorganisms via bidirectional regulation of the IL-17 signaling pathway. However, it was a time-consuming and risky process to draw this kind of conclusion. Analysis of the network had to be up against a problem on how to select the principal results to focus, which is a big challenge but a core in systems pharmacology. Therefore, valid validation of results (e.g., static molecular docking, MD simulation, animals, and biochemistry) is equally important. Through the analysis of this study, IL-17 pathway was found to probably play a critical role in the development and treatment of PID, but relevant research was lacking and incomplete. Hence, serum, integrated pharmacodynamics, and pharmacokinetics will be utilized to clarify the components in serum and relevant therapeutic mechanisms of SCL on PID.

## Figures and Tables

**Table 1:** Docking table with bonding characterization and binding energies in kcal/mol with 14 active compounds involved in five therapeutic targets.

No.	Name	Structure	Glide G-score	MM-GBSA dG bind (kcal/mol)	Bonding interaction	Bond type	Binding protein
4	Polydatin	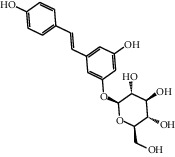	−8.846	−44.64	ASP A 158, GLU A 62, GLU B 360, ASN A 145, SER A 144	H-acc	MAPK1 (PDB ID: 5ax3 native ligand G-score: −8.713)
					TYR A 27, GLN A 45	H-don	
					LYS A 45	Pi-cation	
5	Resveratrol	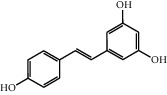	−7.539	−37.18	GLN B 192, SER B 530	H-acc	PTGS2 (PDB ID: 5kir native ligand G-score: −9.800)
					HIE B 90	H-don	
					TRP B 387	Pi-pi stacking	
10	Rutin	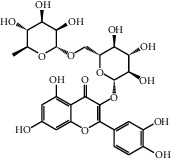	−7.320	−53.30	SER A 68	H-acc	LCN2 (PDB ID: 1x89 native ligand G-score: −7.190)
					ARG A 81	H-don	
					TYR A 106	Pi-pi stacking	
					LYS A 125	Pi-cation	
11	Kaempferol	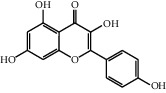	−7.734	−47.73	ALA B 191, GLY B 233	H-acc	MMP9 (PDB ID: 5ue4 native ligand G-score: −8.661)
					HIS B 230	H-don	
					PHE B 110	Pi-pi stacking	
12	Dihydrokaempferol	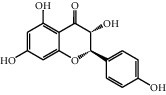	−8.167	−32.82	GLU A 62, ASP A 158, GLU A 24, MET A 99	H-acc	MAPK1
					GLN A 96	H-don	
					LYS A 45	Pi-cation	
17	Quercetin	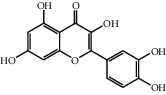	−8.367	−38.06	ASP A 158, GLU B 360, GLU A 62	H-acc	MAPK1
					GLN A 96	H-don	
					LYS A 45	Pi-cation	
18	Isorhamnetin	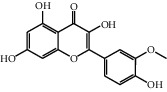	−9.757	−51.06	SER B 530, GLN B 192	H-acc	PTGS2
					PHE B 518, TYR B 385	H-don	
21	Piceatannol	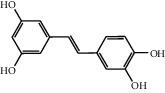	−8.673	−40.61	GLU B 360, GLU A 62, ASP A 158,	H-acc	MAPK1
					GLN A 96	H-don	
					LYS A 45	Pi-cation	
30	Oxyresveratrol	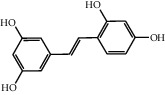	−9.241	−56.71	ALA B 191, HIS B 230, GLY B 233	H-acc	MMP9
					PHE B 110, TYR B 179	Pi-pi stacking	
44	Moracin M	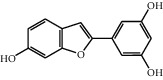	−9.326	−37.01	GLU A 62, GLU B 360, MET A 99	H-acc	MAPK1
					GLN A 96	H-don	
48	Dihydroquercetin	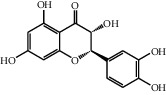	−7.020	−41.83	TYR D 151	H-acc	TNF (PDB ID: 2az5 native ligand G-score: −7.879)
					TYR D 59	Pi-pi stacking	
58	Kaempferide	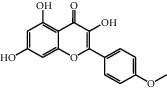	−7.962	−47.23	GLY B 233, ALA B 191	H-acc	MMP9
					HIS B 230	H-don	
					PHE B 110	Pi-pi stacking	
60	Gramine	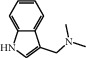	−7.452	−30.51	GLY A 121	H-acc	TNF
62	Dihydrokaempferide	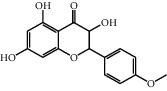	−7.638	−38.65	GLN B 192	H-acc	PTGS2
					TYR B 385	H-don	

MM-GBSA dG bind: the binding energy of the receptor and ligand as calculated by the prime energy, a molecular mechanics + implicit solvent energy. Function (kcal/mol) = prime energy (optimized complex) − prime energy (optimized free ligand) − prime energy (optimized free receptor).

**Table 2:** Time-averaged H-bond properties obtained from MD simulation of five complexes.

Protein	Ligand	H-bond (number)	H-bond distance (average, Å)	H-bond angle (average, °)	Detected H-bonds
PTGS2	Isorhamnetin	2.5	2.94	14.88	18
MAPK1	Moracin M	4.0	2.85	16.15	19
LCN2	Rutin	2.8	2.85	14.59	45
TNF	Rutin	2.2	2.94	16.93	25
MMP9	Oxyresveratrol	4.3	2.78	14.78	20

**Table 3:** Properties of H-bond between five complexes, including occupancy, distance, and angle.

Protein	Donor and acceptor	Occupancy (%)	Distance (Å)	Angle (°)
PTGS2	Phe518 main∼isorhamnetin	73.23	3.06	12.72
	Ile517 main∼isorhamnetin	43.31	2.89	15.01
	Ser530 side∼isorhamnetin	32.92	2.80	14.10

MAPK1	Moracin M∼Glu62 side	124.50	2.82	14.40
	Tyr27 main∼moracin M	67.10	2.90	16.60
	Moracin M∼Asp97 main	63.76	2.74	15.95
	Met99 main∼moracin M	63.29	2.85	16.23

LCN2	Ser68 side∼rutin	63.14	2.77	12.53
	Arg81 side∼Rutin	37.55	2.98	18.48
	Rutin∼Tyr106 side	36.64	2.72	12.10
	Rutin∼Tyr 138 side	35.70	2.69	11.52

TNF	Rutin∼Val123 main	30.74	2.84	13.80
	Rutin∼Tyr119 side	18.45	2.88	16.50
	Ser60-main∼Rutin	17.91	3.03	19.91

MMP9	Oxyresveratrol∼Leu104 main	87.28	2.70	11.43
	Oxyresveratrol∼His230 main	82.36	2.69	14.86
	Oxyresveratrol∼Pro102 main	69.60	2.72	15.57
	Leu234 main∼oxyresveratrol	66.74	2.94	15.81

Occupancy = number of snapshots with H-bond between amino acid residues and ligand during the period of equilibrium/(total number of snapshots during the period of equilibrium (PTGS2:20 ns, MAPK1:30 ns, LCN2:35 ns, TNF:20 ns, MMP9: 25 ns)).

**Table 4:** Molecular mechanics-Poisson Boltzmann surface area (MM-PBSA) binding free energy results for five complexes (kJ mol^−1^).

	PTGS2	MAPK1	LCN2	TNF	MMP9
∆Evdw	−216.440 ± 3.246	−162.988 ± 1.077	−254.221 ± 3.069	−224.329 ± 1.717	−164.405 ± 5.045
∆Eele	−34.967 ± 1.184	−59.667 ± 0.842	−56.01 ± 3.385	−40.788 ± 1.380	−50.800 ± 1.845
∆Epol	122.151 ± 3.055	134.190 ± 1.299	178.273 ± 4.009	180.285 ± 2.108	92.767 ± 3.313
∆Esasa	−17.279 ± 0.243	−14.699 ± 0.075	−23.279 ± 0.144	−22.452 ± 0.171	−13.464 ± 0.391
∆Bind	−146.535 ± 1.934	−103.164 ± 1.533	−155.246 ± 4.579	−107.284 ± 1.485	−135.904 ± 4.201

## Data Availability

The data used to support the findings of the study are included within Supplementary Materials.

## Abbreviations

C-T-P: Compound-target-pathway
∆Eele: Electrostatic energy
∆Epol: Polar solvation interaction
∆Esasa: Solvent-accessible surface area
∆Evdw: van der Waals interaction
GO: Gene Ontology
H-bond: Hydrogen bond
5-HT: 5-Hydroxytryptamine
KEGG: Kyoto Encyclopedia of Genes and Genomes
LCN2: Siderocalin
MAPK1: Mitogen-activated protein kinase 1
MD: Molecular dynamics
MM-GBSA: Molecular mechanics-generalized born surface area
MM-PBSA: Molecular mechanics-Poisson Boltzmann surface area
MMP9: Matrix metalloprotein-9
MMPs: Matrix metalloproteinases
MPO: Myeloperoxidase
NSAIDs: Nonsteroidal anti-inflammatory drugs
NVT: Canonical ensemble
NPT: Constant temperature and pressure
OPLS-2005: Optimum polarized ligand simulation-2005
PME: Particle mesh Ewald
PID: Pelvic inflammatory disease
PPI: Protein-protein interaction
PGE2: Prostaglandin E2
PTGS2: Prostaglandin-endoperoxide synthase 2
RMSD: Root mean square deviation
SCL: *Smilax china* L.
SEA: Similarity ensemble approach
SP: Standard precision
TCM: Traditional Chinese Medicine
TCMID: Traditional Chinese Medicine Integrated Database
TCMSP: Traditional Chinese Medicine System and Pharmacology
TNF: Tumor necrosis factor.
