# Supplementary material for: The Underlying Molecular Mechanisms Involved in Traditional Chinese Medicine Smilax china L. for the Treatment of Pelvic Inflammatory Disease
Source: Evid Based Complement Alternat Med. 2021 Apr 8;2021:5552532. doi: 10.1155/2021/5552532 (PMC8052137; doi:10.1155/2021/5552532)
Supplement: Supplementary Materials — The detailed documents are available in the Supplementary Materials. [file 5552532.f1.docx]

**SUPPLEMENTARY MATERIAL**

**
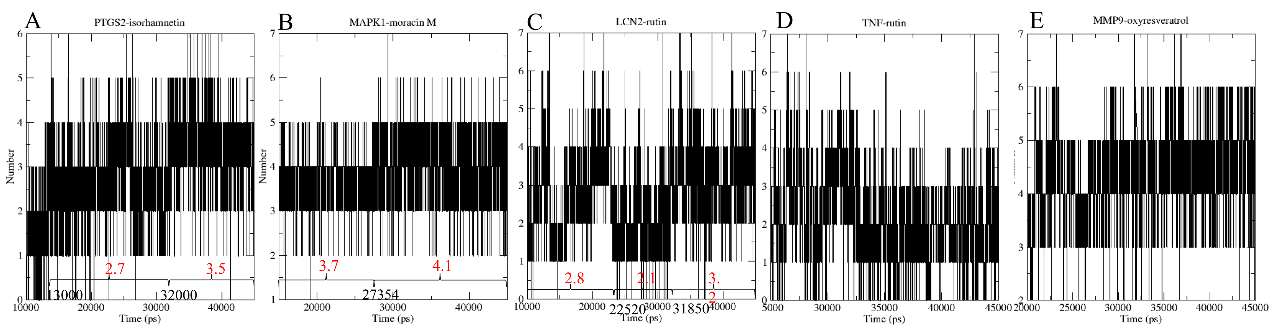
Supplementary Fig. 1.** Total h-bond numbers of five complexes in the fixed time


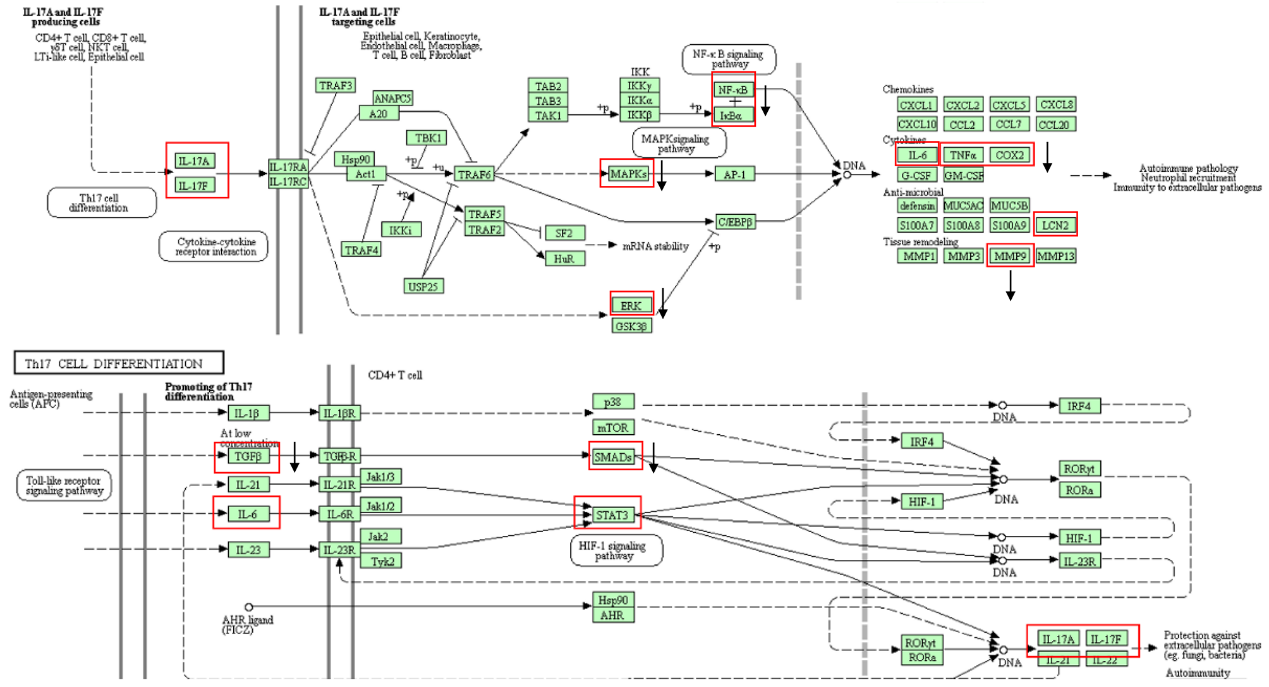


**Supplementary Fig. 2.** IL-17 signaling pathway and Th17 cell differentiation.

**Supplementary Table 1** Parameters setting of five systems.

| Title | Chain | Ions (Na/Cl) | Ions number | Water box | Distance to Box edge (nm) | SOL | Time of MD |
| --- | --- | --- | --- | --- | --- | --- | --- |
| PTGS2 | A, B | Na, Cl | 6, 6 | Dodecahedron | 1.0 | 35597 | 45ns |
| MAPK1 | A, B | Na, Cl | 1, 1 | Cubic | 1.0 | 21833 | 45ns |
| LCN2 | A | Cl | 6 | Cubic | 1.0 | 11144 | 45ns |
| TNF | A, B, C, D | Na | 4 | Dodecahedron | 1.5 | 35773 | 45ns |
| MMP9 | B | Na | 13 | Cubic | 1.0 | 12653 | 45ns |

**Supplementary Table 2** Chemical compounds

| Compounds | Active? | Num. | Structures |
| --- | --- | --- | --- |
| Engeletin | √ | 1 |  |
| Isoengeletin | √ | 2 |  |
| Astilbin | √ | 3 |  |
| Polydatin | √ | 4 |  |
| Resveratrol | √ | 5 |  |
| Kaempferol-7-O-β-D-glu (Populnin) |  | 6 |  |
| Quercetin-3-O-a-L-rhamnoside（Quercetin-3-rhamnoside） |  | 7 |  |
| Sarsasapogenin | √ | 8 |  |
| Dioscin |  | 9 |  |
| Rutin | √ | 10 |  |
| Kaempferol | √ | 11 |  |
| Dihydrokaempferol | √ | 12 |  |
| Kaempferol-5-O-β-D-glucoside |  | 13 |  |
| Quercetin-3'-O-glucoside |  | 14 |  |
| Taxifolin-3-O-glucoside |  | 15 |  |
| Quercetin-4'-O-β-D-glucoside |  | 16 |  |
| quercetin | √ | 17 |  |
| Isorhamnetin | √ | 18 |  |
| Dihydrokaempferol-5-O-β-D-glucoside |  | 19 |  |
| Dihydroquercetin-3-O-α-L-rhamnoside |  | 20 |  |
| Piceatannol | √ | 21 |  |
| 8-[(1R)-1-(3,4-dihydroxyphenyl)-3-methoxy-3-oxopropyl]-substituted catechin 101443796 |  | 22 |  |
| 8-[(1R)-1-(3,4-dihydroxyphenyl)-3-methoxy-3-oxopropyl]-substituted 3-epicatechin |  | 23 |  |
| 6-[(1S)-3-methoxy-3-oxo-1-(2,4,5-trihydroxyphe-nyl) propyl]- substituted catechin |  | 24 |  |
| 6-[(1R)-3-methoxy-3-oxo-1-(2,4,5-trihydroxyphe-nyl) propyl]-substituted catechin |  | 25 |  |
| (2R,3R)-Dihydrokaempferol-3-O-(6"-oxy-acetyl)-β-D-glucoside |  | 26 |  |
| (2R,3R)-Dihydrokaempferol-3-O-(2"-oxy-acetyl)-β-D-glucoside |  | 27 |  |
| (2R,3R)-Dihydrokaempferol-3-O-(3"-oxy-acetyl)-β-D-glucoside |  | 28 |  |
| (2R,3R)-Dihydrokaempferol-3-O-β-D-glucoside |  | 29 |  |
| Oxyresveratrol | √ | 30 |  |
| β-sitosterol |  | 31 |  |
| Daucosterol |  | 32 |  |
| Saponin A |  | 33 |  |
| Saponin B |  | 34 |  |
| Saponin C |  | 35 |  |
| Saponin D |  | 36 |  |
| Butyl (Z)-3-(3,4-dihydroxyphenyl) prop-2-enoate |  | 37 |  |
| Butyl β-D-fructopyranoside | √ | 38 |  |
| Syringic acid | √ | 39 |  |
| Protocatechuic acid | √ | 40 |  |
| Vanillic acid | √ | 41 |  |
| 3,5-Dimethoxy-4-β-D-glucopyranosyl cinnamic acid | √ | 42 |  |
| Maackoline | √ | 43 |  |
| Moracin M | √ | 44 |  |
| Gallic acid | √ | 45 |  |
| Trans-2-hydroxycinnamic acid | √ | 46 |  |
| Gentisic acid | √ | 47 |  |
| Dihydroquercetin | √ | 48 |  |
| Dihydroquercetin-3'-O-glucoside |  | 49 |  |
| Oleanolic acid | √ | 50 |  |
| Caffeic acid | √ | 51 |  |
| Ergosterol |  | 52 |  |
| Methylprotodioscin_qt | √ | 53 |  |
| Pseudodiosgenin |  | 54 |  |
| Coumarin | √ | 55 |  |
| Methylprotodioscin |  | 56 |  |
| Methylprotogracillin |  | 57 |  |
| Kaempferide | √ | 58 |  |
| Pseudoprotodioscin |  | 59 |  |
| Gramine | √ | 60 |  |
| Scirpusin A |  | 61 |  |
| Dihydrokaempferide | √ | 62 |  |
| Diosgenin | √ | 63 |  |
| Palmitic acid |  | 64 |  |
| Chlorogenic acid |  | 65 |  |
| Parillin |  | 66 |  |
| S. aristolochiaefolia mill |  | 67 |  |
| Smilaxin |  | 68 |  |

**Supplementary Table 3** 718 candidates targets of 32 active compounds

| Title | ID |
| --- | --- |
| Engeletin | 6PGD |
| Engeletin | CAH3 |
| Engeletin | CAH4 |
| Engeletin | CAH7 |
| Engeletin | CBR1 |
| Engeletin | CP1B1 |
| Engeletin | ESR1 |
| Engeletin | ESR2 |
| Engeletin | KLK2 |
| Engeletin | NMUR2 |
| Engeletin | PDIA1 |
| Engeletin | T2R31 |
| Engeletin | PGH1 |
| Engeletin | CP19A |
| Engeletin | SC5A1 |
| Engeletin | SC5A4 |
| Engeletin | SC5A2 |
| Engeletin | CAH12 |
| Engeletin | S28A3 |
| Engeletin | TYRO |
| Engeletin | CAH2 |
| Engeletin | CAH1 |
| Engeletin | MMP13 |
| Engeletin | MMP12 |
| Engeletin | RASH |
| Engeletin | TTHY |
| Engeletin | PTN1 |
| Engeletin | MK10 |
| Engeletin | PPIA |
| Engeletin | NR1H2 |
| Engeletin | CDK5 |
| Engeletin | THRB |
| Engeletin | CMA1 |
| Engeletin | CFAB |
| Engeletin | LEG7 |
| Engeletin | PIM1 |
| Engeletin | SAMP |
| Engeletin | LV208 |
| Engeletin | MK08 |
| Engeletin | GSTP1 |
| Engeletin | CAH2 |
| Engeletin | DAPK1 |
| Engeletin | CDK2 |
| Engeletin | EGFR |
| Engeletin | TYRO |
| Engeletin | MK01 |
| Isoengeletin | 6PGD |
| Isoengeletin | CAH3 |
| Isoengeletin | CAH4 |
| Isoengeletin | CAH7 |
| Isoengeletin | CBR1 |
| Isoengeletin | CP1B1 |
| Isoengeletin | ESR1 |
| Isoengeletin | ESR2 |
| Isoengeletin | KLK2 |
| Isoengeletin | NMUR2 |
| Isoengeletin | PDIA1 |
| Isoengeletin | T2R31 |
| Isoengeletin | PGH1 |
| Isoengeletin | CP19A |
| Isoengeletin | SC5A1 |
| Isoengeletin | SC5A4 |
| Isoengeletin | SC5A2 |
| Isoengeletin | S28A3 |
| Isoengeletin | ALDR |
| Isoengeletin | CAH12 |
| Isoengeletin | MMP13 |
| Isoengeletin | MMP12 |
| Isoengeletin | TYRO |
| Isoengeletin | TOP1 |
| Isoengeletin | MMP2 |
| Isoengeletin | CAH2 |
| Isoengeletin | THRB |
| Isoengeletin | PPIA |
| Isoengeletin | ANDR |
| Isoengeletin | CFAB |
| Isoengeletin | SAMP |
| Isoengeletin | EEA1 |
| Isoengeletin | NR1H2 |
| Isoengeletin | GSTP1 |
| Isoengeletin | CAH2 |
| Isoengeletin | ALDR |
| Isoengeletin | PTN1 |
| Isoengeletin | TTHY |
| Isoengeletin | FABP4 |
| Isoengeletin | MK08 |
| Isoengeletin | PPARG |
| Isoengeletin | SHBG |
| Isoengeletin | PIM1 |
| Isoengeletin | FOLH1 |
| Isoengeletin | PDE4B |
| Isoengeletin | RXRA |
| Isoengeletin | TYRO |
| Astilbin | 6PGD |
| Astilbin | CAH3 |
| Astilbin | PGH1 |
| Astilbin | CP19A |
| Astilbin | MMP13 |
| Astilbin | MMP12 |
| Astilbin | CAH12 |
| Astilbin | MMP2 |
| Astilbin | CAH2 |
| Astilbin | CAH1 |
| Astilbin | CAH6 |
| Astilbin | CAH5B |
| Astilbin | SC5A1 |
| Astilbin | SC5A4 |
| Astilbin | SC5A2 |
| Astilbin | TAU |
| Astilbin | TREM1 |
| Astilbin | PTN1 |
| Astilbin | THRB |
| Astilbin | PIM1 |
| Astilbin | ALBU |
| Astilbin | MIF |
| Astilbin | CDK2 |
| Astilbin | CAH2 |
| Astilbin | CHK1 |
| Astilbin | OTC |
| Astilbin | SRC |
| Astilbin | CATB |
| Astilbin | FGF1 |
| Astilbin | ALDR |
| Astilbin | GLRX1 |
| Astilbin | EGFR |
| Astilbin | GSK3B |
| Astilbin | PH4H |
| Astilbin | GSHB |
| Astilbin | UROK |
| Astilbin | CAH4 |
| Astilbin | CAH7 |
| Astilbin | CBR1 |
| Astilbin | CP1B1 |
| Astilbin | CP2C8 |
| Astilbin | ELAV1 |
| Astilbin | KLK2 |
| Astilbin | NMUR2 |
| Astilbin | PDIA1 |
| Astilbin | PLGF |
| Astilbin | RASH |
| Astilbin | T2R31 |
| Astilbin | VEGFA |
| Polydatin | AL1A2 |
| Polydatin | ALDR |
| Polydatin | TYRO |
| Polydatin | TNFA |
| Polydatin | SC5A1 |
| Polydatin | AA2AR |
| Polydatin | MMP13 |
| Polydatin | MMP1 |
| Polydatin | MMP7 |
| Polydatin | MMP12 |
| Polydatin | MMP8 |
| Polydatin | PGH1 |
| Polydatin | PGH2 |
| Polydatin | IL2 |
| Polydatin | SC5A4 |
| Polydatin | AL1B1 |
| Polydatin | PPIA |
| Polydatin | ANDR |
| Polydatin | BMP2 |
| Polydatin | GSTP1 |
| Polydatin | NGAL |
| Polydatin | PIM1 |
| Polydatin | ALDR |
| Polydatin | TTHY |
| Polydatin | PDE5A |
| Polydatin | CP19A |
| Polydatin | CAH2 |
| Polydatin | RXRA |
| Polydatin | CDK2 |
| Polydatin | PTN1 |
| Polydatin | ESR1 |
| Polydatin | TYSY |
| Polydatin | EPHB4 |
| Polydatin | ALBU |
| Polydatin | GSHR |
| Polydatin | THRB |
| Polydatin | MAOM |
| Polydatin | PRGR |
| Polydatin | MAP2 |
| Polydatin | ACE |
| Polydatin | ADHX |
| Polydatin | EGFR |
| Polydatin | ANXA5 |
| Polydatin | BRAF |
| Polydatin | MET |
| Polydatin | MCR |
| Polydatin | FABP4 |
| Polydatin | SAMP |
| Polydatin | NOS3 |
| Polydatin | PPP5 |
| Polydatin | ERR3 |
| Polydatin | CHK1 |
| Polydatin | EEA1 |
| Polydatin | DUS6 |
| Polydatin | ADA17 |
| Polydatin | AMYP |
| Polydatin | B4GT1 |
| Polydatin | CAH13 |
| Polydatin | CAH14 |
| Polydatin | CAH12 |
| Polydatin | CAH2 |
| Polydatin | CAH1 |
| Polydatin | CAH4 |
| Polydatin | CAH7 |
| Polydatin | CAH9 |
| Polydatin | CP1B1 |
| Polydatin | DCOR |
| Polydatin | ERAP1 |
| Polydatin | FGF1 |
| Polydatin | FGF2 |
| Polydatin | HPSE |
| Polydatin | IL2 |
| Polydatin | LEG3 |
| Polydatin | LEG4 |
| Polydatin | LEG8 |
| Polydatin | LEG9 |
| Polydatin | MGMT |
| Polydatin | NMUR2 |
| Polydatin | PDIA1 |
| Polydatin | RASH |
| Polydatin | RASN |
| Polydatin | S28A3 |
| Polydatin | SC5A1 |
| Polydatin | SC5A2 |
| Polydatin | SC5A4 |
| Polydatin | SC5AB |
| Polydatin | TOP1 |
| Polydatin | TYRO |
| Polydatin | VEGFA |
| Polydatin | MK01 |
| Resveratrol | A4 |
| Resveratrol | MK01 |
| Resveratrol | AOFA |
| Resveratrol | CAH2 |
| Resveratrol | ESR1 |
| Resveratrol | PGH1 |
| Resveratrol | SC6A2 |
| Resveratrol | PGH2 |
| Resveratrol | CAH7 |
| Resveratrol | A4 |
| Resveratrol | CAH1 |
| Resveratrol | CAH3 |
| Resveratrol | CAH6 |
| Resveratrol | PK3CB |
| Resveratrol | CAH12 |
| Resveratrol | CP1A2 |
| Resveratrol | CP2C9 |
| Resveratrol | CP3A4 |
| Resveratrol | CAH14 |
| Resveratrol | CAH9 |
| Resveratrol | CP2CJ |
| Resveratrol | CAH4 |
| Resveratrol | CAH13 |
| Resveratrol | NQO2 |
| Resveratrol | CAH5B |
| Resveratrol | PK3CA |
| Resveratrol | CAH5A |
| Resveratrol | LCK |
| Resveratrol | KSYK |
| Resveratrol | BMP2 |
| Resveratrol | ALDR |
| Resveratrol | KDM4E |
| Resveratrol | PTN1 |
| Resveratrol | THRB |
| Resveratrol | MAP2 |
| Resveratrol | PPP5 |
| Resveratrol | DUS6 |
| Resveratrol | ALBU |
| Resveratrol | CAH2 |
| Resveratrol | CHLE |
| Resveratrol | CDK2 |
| Resveratrol | TTHY |
| Resveratrol | GLRX1 |
| Resveratrol | CTNA1 |
| Resveratrol | SRC |
| Resveratrol | PLGF |
| Resveratrol | MK14 |
| Resveratrol | MMP8 |
| Resveratrol | PRGR |
| Resveratrol | ABCG2 |
| Resveratrol | AHR |
| Resveratrol | AK1BA |
| Resveratrol | ALR |
| Resveratrol | AOFA |
| Resveratrol | AOFB |
| Resveratrol | BACE1 |
| Resveratrol | CAH13 |
| Resveratrol | CAH14 |
| Resveratrol | CAH12 |
| Resveratrol | CAH1 |
| Resveratrol | CAH3 |
| Resveratrol | CAH4 |
| Resveratrol | CAH2 |
| Resveratrol | CAH5B |
| Resveratrol | CAH5A |
| Resveratrol | CAH7 |
| Resveratrol | CAH6 |
| Resveratrol | CAH9 |
| Resveratrol | CISD1 |
| Resveratrol | CP1A1 |
| Resveratrol | CP1B1 |
| Resveratrol | CP1A2 |
| Resveratrol | CP2C9 |
| Resveratrol | CP2CJ |
| Resveratrol | CP3A4 |
| Resveratrol | DCOR |
| Resveratrol | ESR1 |
| Resveratrol | ESR2 |
| Resveratrol | G6PC |
| Resveratrol | GABT |
| Resveratrol | KCC2A |
| Resveratrol | KKCC2 |
| Resveratrol | LGUL |
| Resveratrol | LOX5 |
| Resveratrol | M3K10 |
| Resveratrol | MARK4 |
| Resveratrol | MIF |
| Resveratrol | NF2L2 |
| Resveratrol | NQO2 |
| Resveratrol | PGH1 |
| Resveratrol | PGH2 |
| Resveratrol | PK3CB |
| Resveratrol | PK3CA |
| Resveratrol | PTN6 |
| Resveratrol | SC6A2 |
| Resveratrol | SIK2 |
| Resveratrol | SSDH |
| Resveratrol | TAU |
| Resveratrol | TBB1 |
| Resveratrol | TBB3 |
| Resveratrol | TF65 |
| Resveratrol | TF |
| Resveratrol | TRPA1 |
| Resveratrol | TTHY |
| Sarsasapogenin | AT12A |
| Sarsasapogenin | CBG |
| Sarsasapogenin | PSN2 |
| Sarsasapogenin | CP51A |
| Sarsasapogenin | HMDH |
| Sarsasapogenin | HS90A |
| Sarsasapogenin | NR1H3 |
| Sarsasapogenin | SHH |
| Sarsasapogenin | NPCL1 |
| Sarsasapogenin | EST2 |
| Sarsasapogenin | DHI1 |
| Sarsasapogenin | NOS2 |
| Sarsasapogenin | AK1C3 |
| Sarsasapogenin | ERG7 |
| Sarsasapogenin | ALK |
| Sarsasapogenin | PDE10 |
| Sarsasapogenin | NR1H4 |
| Sarsasapogenin | IF4A1 |
| Sarsasapogenin | MAPK2 |
| Sarsasapogenin | AK1C2 |
| Sarsasapogenin | KIF11 |
| Sarsasapogenin | PIM1 |
| Sarsasapogenin | MK01 |
| Sarsasapogenin | CASP7 |
| Sarsasapogenin | ANDR |
| Sarsasapogenin | STS |
| Sarsasapogenin | ALBU |
| Sarsasapogenin | ITAL |
| Sarsasapogenin | BMP2 |
| Sarsasapogenin | CFAB |
| Sarsasapogenin | MK10 |
| Sarsasapogenin | AOFB |
| Sarsasapogenin | MK14 |
| Sarsasapogenin | CAH2 |
| Sarsasapogenin | TTHY |
| Sarsasapogenin | PK3CG |
| Sarsasapogenin | VTDB |
| Sarsasapogenin | IL2 |
| Sarsasapogenin | NMDE2 |
| Sarsasapogenin | NMDE3 |
| Sarsasapogenin | NMDE4 |
| Sarsasapogenin | NMDZ1 |
| Sarsasapogenin | SHBG |
| Sarsasapogenin | SIA4A |
| Sarsasapogenin | 3MG |
| Sarsasapogenin | ABCG2 |
| Sarsasapogenin | ACES |
| Sarsasapogenin | ADA2A |
| Sarsasapogenin | ADA2C |
| Rutin | AK1BA |
| Rutin | AL1A2 |
| Rutin | AL1B1 |
| Rutin | NMUR2 |
| Rutin | ADA2A |
| Rutin | ADA2C |
| Rutin | ALDR |
| Rutin | CAH7 |
| Rutin | CAH12 |
| Rutin | CAH4 |
| Rutin | NOX4 |
| Rutin | CAH2 |
| Rutin | NQO2 |
| Rutin | KS6A3 |
| Rutin | PGH2 |
| Rutin | ALDR |
| Rutin | MK10 |
| Rutin | GSTP1 |
| Rutin | PNPH |
| Rutin | MK08 |
| Rutin | CFAB |
| Rutin | THRB |
| Rutin | TREM1 |
| Rutin | MAOM |
| Rutin | CHK1 |
| Rutin | ALDR |
| Rutin | CMA1 |
| Rutin | FA10 |
| Rutin | TGFR1 |
| Rutin | SERB |
| Rutin | SRC |
| Rutin | PTN1 |
| Rutin | KIF11 |
| Rutin | FOLH1 |
| Rutin | LEG7 |
| Rutin | CATB |
| Rutin | CAH12 |
| Rutin | CAH4 |
| Rutin | CAH7 |
| Rutin | CBR1 |
| Rutin | CP1B1 |
| Rutin | CP2C8 |
| Rutin | DAPK1 |
| Rutin | ELAV1 |
| Rutin | ERAP1 |
| Rutin | GPR35 |
| Rutin | IL2 |
| Rutin | KCND3 |
| Rutin | LGUL |
| Rutin | LOX5 |
| Rutin | MDR1 |
| Rutin | MRP1 |
| Rutin | NEK6 |
| Rutin | NMUR2 |
| Rutin | NOX4 |
| Rutin | PDIA1 |
| Rutin | PKN1 |
| Rutin | PPBI |
| Rutin | PTPRS |
| Rutin | S28A3 |
| Rutin | SIAT1 |
| Rutin | TYRO |
| Rutin | XDH |
| Rutin | 3MG |
| Rutin | ABCG2 |
| Rutin | AHR |
| Rutin | AK1BA |
| Rutin | ALDH2 |
| Kaempferol | ALDR |
| Kaempferol | AMY1 |
| Kaempferol | NOX4 |
| Kaempferol | ALDR |
| Kaempferol | XDH |
| Kaempferol | TYRO |
| Kaempferol | FLT3 |
| Kaempferol | CAH2 |
| Kaempferol | LOX5 |
| Kaempferol | CAH7 |
| Kaempferol | DHB2 |
| Kaempferol | DHB1 |
| Kaempferol | AHR |
| Kaempferol | CAH12 |
| Kaempferol | ERR1 |
| Kaempferol | CP1B1 |
| Kaempferol | CAH4 |
| Kaempferol | ACES |
| Kaempferol | AOFA |
| Kaempferol | LGUL |
| Kaempferol | KSYK |
| Kaempferol | GSK3B |
| Kaempferol | MMP9 |
| Kaempferol | MMP2 |
| Kaempferol | LOX15 |
| Kaempferol | LOX12 |
| Kaempferol | PTPRS |
| Kaempferol | AA2AR |
| Kaempferol | ANDR |
| Kaempferol | SNRPA |
| Kaempferol | PTN1 |
| Kaempferol | HEM2 |
| Kaempferol | FOLH1 |
| Kaempferol | HMDH |
| Kaempferol | SRC |
| Kaempferol | GLRX1 |
| Kaempferol | CBR1 |
| Kaempferol | UMPS |
| Kaempferol | PCKGC |
| Kaempferol | LDHB |
| Kaempferol | PYRD |
| Kaempferol | HPGDS |
| Kaempferol | PPAP |
| Kaempferol | DYR |
| Kaempferol | MAPK2 |
| Kaempferol | XIAP |
| Kaempferol | ACE |
| Kaempferol | GSTT2 |
| Kaempferol | ALDR |
| Kaempferol | AOFA |
| Kaempferol | APEX1 |
| Kaempferol | CAH12 |
| Kaempferol | CAH3 |
| Kaempferol | CAH2 |
| Kaempferol | CAH4 |
| Kaempferol | CAH7 |
| Kaempferol | CALM1 |
| Kaempferol | CBR1 |
| Kaempferol | CCR4 |
| Kaempferol | CISD1 |
| Kaempferol | CP1B1 |
| Kaempferol | CP1A2 |
| Kaempferol | CP2C8 |
| Kaempferol | CP2D6 |
| Kaempferol | CREB1 |
| Kaempferol | CTDS1 |
| Kaempferol | DAPK1 |
| Kaempferol | DHB1 |
| Kaempferol | DHB2 |
| Kaempferol | DHB3 |
| Kaempferol | ELAV1 |
| Kaempferol | ERAP1 |
| Kaempferol | ERR1 |
| Kaempferol | ERR2 |
| Kaempferol | ESR1 |
| Kaempferol | ESR2 |
| Kaempferol | FLT3 |
| Kaempferol | GBG2 |
| Kaempferol | GBB1 |
| Kaempferol | GPR35 |
| Kaempferol | GRK6 |
| Kaempferol | GTR1 |
| Kaempferol | IL2 |
| Kaempferol | KCND3 |
| Kaempferol | KDM4E |
| Kaempferol | LGUL |
| Kaempferol | LOX12 |
| Kaempferol | LOX15 |
| Kaempferol | LOX5 |
| Kaempferol | MDR1 |
| Kaempferol | MIF |
| Kaempferol | MRP1 |
| Kaempferol | NEK6 |
| Kaempferol | NMUR2 |
| Kaempferol | NOX4 |
| Kaempferol | NQO1 |
| Kaempferol | NUAK1 |
| Kaempferol | PDIA1 |
| Kaempferol | PERM |
| Kaempferol | PKN1 |
| Kaempferol | PLGF |
| Kaempferol | PON1 |
| Kaempferol | PPAC |
| Kaempferol | PPBI |
| Kaempferol | PTPRS |
| Kaempferol | SIAT1 |
| Kaempferol | TAU |
| Kaempferol | TERT |
| Kaempferol | TNKS1 |
| Kaempferol | TNKS2 |
| Kaempferol | TOP2A |
| Kaempferol | TTHY |
| Kaempferol | TYRO |
| Kaempferol | VEGFA |
| Kaempferol | XDH |
| Kaempferol | 6PGD |
| Kaempferol | CAH3 |
| Kaempferol | CAH4 |
| Kaempferol | CAH7 |
| Kaempferol | CBR1 |
| Dihydrokaempferol | 6PGD |
| Dihydrokaempferol | MK01 |
| Dihydrokaempferol | CAH3 |
| Dihydrokaempferol | CAH4 |
| Dihydrokaempferol | CAH7 |
| Dihydrokaempferol | CBR1 |
| Dihydrokaempferol | CP1B1 |
| Dihydrokaempferol | ESR1 |
| Dihydrokaempferol | ESR2 |
| Dihydrokaempferol | FUT4 |
| Dihydrokaempferol | FUT7 |
| Dihydrokaempferol | KLK2 |
| Dihydrokaempferol | MRP2 |
| Dihydrokaempferol | PLGF |
| Dihydrokaempferol | SIAT6 |
| Dihydrokaempferol | T2R31 |
| Dihydrokaempferol | VEGFA |
| Dihydrokaempferol | PDE5A |
| Dihydrokaempferol | SNRPA |
| Dihydrokaempferol | FOLH1 |
| Dihydrokaempferol | SRC |
| Dihydrokaempferol | GLRX1 |
| Dihydrokaempferol | CBR1 |
| Dihydrokaempferol | LDHB |
| Dihydrokaempferol | PTN1 |
| Dihydrokaempferol | UMPS |
| Dihydrokaempferol | UCK2 |
| Dihydrokaempferol | PYRD |
| Dihydrokaempferol | PPAP |
| Dihydrokaempferol | PCKGC |
| Dihydrokaempferol | HPGDS |
| Dihydrokaempferol | XIAP |
| Dihydrokaempferol | GSTT2 |
| Dihydrokaempferol | INSR |
| Dihydrokaempferol | HCK |
| Dihydrokaempferol | THRB |
| Dihydrokaempferol | ALDR |
| Dihydrokaempferol | XDH |
| Quercetin | 3MG |
| Quercetin | MK01 |
| Quercetin | ABCG2 |
| Quercetin | AK1BA |
| Quercetin | AKT1 |
| Quercetin | ALDR |
| Quercetin | ALK |
| Quercetin | AMY1 |
| Quercetin | AOFA |
| Quercetin | APEX1 |
| Quercetin | AURKB |
| Quercetin | BACE1 |
| Quercetin | CAH14 |
| Quercetin | CAH12 |
| Quercetin | CAH3 |
| Quercetin | CAH2 |
| Quercetin | CAH1 |
| Quercetin | CAH4 |
| Quercetin | CAH5A |
| Quercetin | CAH6 |
| Quercetin | CAH7 |
| Quercetin | CAH9 |
| Quercetin | CBR1 |
| Quercetin | CCR4 |
| Quercetin | CDK1 |
| Quercetin | CISD1 |
| Quercetin | CP1B1 |
| Quercetin | CP1A2 |
| Quercetin | CP2C8 |
| Quercetin | CP2C9 |
| Quercetin | CREB1 |
| Quercetin | CSK21 |
| Quercetin | CXCR1 |
| Quercetin | DAPK1 |
| Quercetin | DHB1 |
| Quercetin | DHB2 |
| Quercetin | DHPR |
| Quercetin | DRD4 |
| Quercetin | EGFR |
| Quercetin | ELAV1 |
| Quercetin | ERR1 |
| Quercetin | ERR2 |
| Quercetin | ESR1 |
| Quercetin | ESR2 |
| Quercetin | FAK1 |
| Quercetin | FLT3 |
| Quercetin | GPR35 |
| Quercetin | GRK6 |
| Quercetin | GSK3B |
| Quercetin | IGF1R |
| Quercetin | IL2 |
| Quercetin | KCC2B |
| Quercetin | KCND3 |
| Quercetin | KDM4E |
| Quercetin | LGUL |
| Quercetin | LOX12 |
| Quercetin | LOX15 |
| Quercetin | LOX5 |
| Quercetin | MDR1 |
| Quercetin | MET |
| Quercetin | MMP13 |
| Quercetin | MMP2 |
| Quercetin | MMP3 |
| Quercetin | MMP9 |
| Quercetin | MRP1 |
| Quercetin | NEK2 |
| Quercetin | NEK6 |
| Quercetin | NMUR2 |
| Quercetin | NOX4 |
| Quercetin | NUAK1 |
| Quercetin | P85A |
| Quercetin | PA21B |
| Quercetin | PDIA1 |
| Quercetin | PERM |
| Quercetin | PIM1 |
| Quercetin | PKN1 |
| Quercetin | PLGF |
| Quercetin | PON1 |
| Quercetin | PPAC |
| Quercetin | PPBI |
| Quercetin | PTPRS |
| Quercetin | PYGL |
| Quercetin | SIAT1 |
| Quercetin | SYUA |
| Quercetin | TAU |
| Quercetin | TERT |
| Quercetin | THRB |
| Quercetin | TOP2A |
| Quercetin | TTHY |
| Quercetin | TYRO |
| Quercetin | UFO |
| Quercetin | V2R |
| Quercetin | VEGFA |
| Quercetin | VGFR2 |
| Quercetin | NOX4 |
| Quercetin | V2R |
| Quercetin | ALDR |
| Quercetin | XDH |
| Quercetin | AOFA |
| Quercetin | IGF1R |
| Quercetin | FLT3 |
| Quercetin | CP19A |
| Quercetin | EGFR |
| Quercetin | THRB |
| Quercetin | CAH2 |
| Quercetin | PIM1 |
| Quercetin | LOX5 |
| Quercetin | AURKB |
| Quercetin | DRD4 |
| Quercetin | AA1R |
| Quercetin | CAH7 |
| Quercetin | LGUL |
| Quercetin | PERM |
| Quercetin | P85A |
| Quercetin | AA2AR |
| Quercetin | DAPK1 |
| Quercetin | PYGL |
| Quercetin | CAH1 |
| Quercetin | GSK3B |
| Quercetin | SRC |
| Quercetin | FAK1 |
| Quercetin | DHB2 |
| Quercetin | VGFR2 |
| Quercetin | MMP13 |
| Quercetin | MMP3 |
| Quercetin | CAH3 |
| Quercetin | LOX15 |
| Quercetin | PLK1 |
| Quercetin | CAH6 |
| Quercetin | CDK1 |
| Quercetin | MMP9 |
| Quercetin | CAH12 |
| Quercetin | MMP2 |
| Quercetin | PKN1 |
| Quercetin | CAH14 |
| Quercetin | CAH9 |
| Quercetin | CSK21 |
| Quercetin | LOX12 |
| Quercetin | MET |
| Quercetin | CAH4 |
| Quercetin | NEK2 |
| Quercetin | CXCR1 |
| Quercetin | KCC2B |
| Quercetin | ALK |
| Quercetin | AKT1 |
| Quercetin | NEK6 |
| Quercetin | PA21B |
| Quercetin | CAH5A |
| Quercetin | BACE1 |
| Quercetin | CP1B1 |
| Quercetin | UFO |
| Quercetin | ABCG2 |
| Quercetin | NUAK1 |
| Quercetin | AK1C2 |
| Quercetin | AK1C1 |
| Quercetin | AK1C3 |
| Quercetin | AK1C4 |
| Quercetin | CAH13 |
| Quercetin | AK1A1 |
| Quercetin | GPR35 |
| Quercetin | SNRPA |
| Quercetin | PTN1 |
| Quercetin | HEM2 |
| Quercetin | HMDH |
| Quercetin | SRC |
| Quercetin | FGF1 |
| Quercetin | FOLH1 |
| Quercetin | GLRX1 |
| Quercetin | PCKGC |
| Quercetin | CBR1 |
| Quercetin | ACE |
| Quercetin | RNAS4 |
| Quercetin | UMPS |
| Quercetin | GSHR |
| Quercetin | DYR |
| Quercetin | LDHB |
| Quercetin | PYRD |
| Quercetin | HPGDS |
| Quercetin | PPAP |
| Quercetin | XDH |
| Isorhamnetin | 3MG |
| Isorhamnetin | A4 |
| Isorhamnetin | ABCG2 |
| Isorhamnetin | AK1BA |
| Isorhamnetin | ALDR |
| Isorhamnetin | AMY1 |
| Isorhamnetin | AOFA |
| Isorhamnetin | APEX1 |
| Isorhamnetin | BRAF |
| Isorhamnetin | CAH13 |
| Isorhamnetin | CAH12 |
| Isorhamnetin | CAH3 |
| Isorhamnetin | CAH4 |
| Isorhamnetin | CAH2 |
| Isorhamnetin | CAH5A |
| Isorhamnetin | CAH6 |
| Isorhamnetin | CAH7 |
| Isorhamnetin | CBR1 |
| Isorhamnetin | CCND3 |
| Isorhamnetin | CCR4 |
| Isorhamnetin | CHK1 |
| Isorhamnetin | CISD1 |
| Isorhamnetin | CP1B1 |
| Isorhamnetin | CP2C8 |
| Isorhamnetin | CREB1 |
| Isorhamnetin | DAPK1 |
| Isorhamnetin | DCOR |
| Isorhamnetin | DHB2 |
| Isorhamnetin | DHPR |
| Isorhamnetin | DYRK4 |
| Isorhamnetin | ELAV1 |
| Isorhamnetin | EP300 |
| Isorhamnetin | ERAP1 |
| Isorhamnetin | ERN1 |
| Isorhamnetin | ERR1 |
| Isorhamnetin | ESR1 |
| Isorhamnetin | ESR2 |
| Isorhamnetin | FOS |
| Isorhamnetin | GPR35 |
| Isorhamnetin | GRK6 |
| Isorhamnetin | IL2 |
| Isorhamnetin | ITF2 |
| Isorhamnetin | KCC2A |
| Isorhamnetin | KCC2B |
| Isorhamnetin | KCND3 |
| Isorhamnetin | KDM4E |
| Isorhamnetin | LGUL |
| Isorhamnetin | LOX12 |
| Isorhamnetin | LOX15 |
| Isorhamnetin | LOX5 |
| Isorhamnetin | LRP6 |
| Isorhamnetin | MDR1 |
| Isorhamnetin | MRP1 |
| Isorhamnetin | NEK6 |
| Isorhamnetin | NFKB1 |
| Isorhamnetin | NF2L2 |
| Isorhamnetin | NMUR2 |
| Isorhamnetin | NOX4 |
| Isorhamnetin | NQO1 |
| Isorhamnetin | NUAK1 |
| Isorhamnetin | PDIA1 |
| Isorhamnetin | PERM |
| Isorhamnetin | PKN1 |
| Isorhamnetin | PLGF |
| Isorhamnetin | PPAC |
| Isorhamnetin | PPBI |
| Isorhamnetin | PTPRS |
| Isorhamnetin | RAF1 |
| Isorhamnetin | SHBG |
| Isorhamnetin | SIAT1 |
| Isorhamnetin | T2R31 |
| Isorhamnetin | TAU |
| Isorhamnetin | TERT |
| Isorhamnetin | TTHY |
| Isorhamnetin | TYRO |
| Isorhamnetin | VEGFA |
| Isorhamnetin | XDH |
| Isorhamnetin | CAH2 |
| Isorhamnetin | CAH7 |
| Isorhamnetin | CAH12 |
| Isorhamnetin | CAH4 |
| Isorhamnetin | CP1B1 |
| Isorhamnetin | NOX4 |
| Isorhamnetin | ALDR |
| Isorhamnetin | ANGI |
| Isorhamnetin | ALDR |
| Isorhamnetin | SNRPA |
| Isorhamnetin | CAH2 |
| Isorhamnetin | BCAT2 |
| Isorhamnetin | CDK2 |
| Isorhamnetin | PTN1 |
| Isorhamnetin | PDPK1 |
| Isorhamnetin | HEM2 |
| Isorhamnetin | PDE4B |
| Isorhamnetin | CSK21 |
| Isorhamnetin | MIF |
| Isorhamnetin | SRC |
| Isorhamnetin | HMDH |
| Isorhamnetin | FOLH1 |
| Isorhamnetin | CCNA2 |
| Isorhamnetin | GLRX1 |
| Isorhamnetin | PDE4D |
| Isorhamnetin | AK1C3 |
| Isorhamnetin | UMPS |
| Isorhamnetin | XDH |
| Piceatannol | A4 |
| Piceatannol | MK01 |
| Piceatannol | ABCG2 |
| Piceatannol | ACK1 |
| Piceatannol | ACVR1 |
| Piceatannol | AHR |
| Piceatannol | AK1BA |
| Piceatannol | AK1C4 |
| Piceatannol | ALDR |
| Piceatannol | ALK |
| Piceatannol | ALR |
| Piceatannol | BTK |
| Piceatannol | CAH13 |
| Piceatannol | CAH14 |
| Piceatannol | CAH3 |
| Piceatannol | CAH4 |
| Piceatannol | CAH5A |
| Piceatannol | CAH5B |
| Piceatannol | CAH6 |
| Piceatannol | CAH7 |
| Piceatannol | CASP6 |
| Piceatannol | CDK8 |
| Piceatannol | CH60 |
| Piceatannol | CISD1 |
| Piceatannol | CLK2 |
| Piceatannol | CP1A1 |
| Piceatannol | CP1B1 |
| Piceatannol | CP1A2 |
| Piceatannol | DAPK2 |
| Piceatannol | DCOR |
| Piceatannol | DHPR |
| Piceatannol | DYN1 |
| Piceatannol | DYR1A |
| Piceatannol | DYR1B |
| Piceatannol | EP300 |
| Piceatannol | FAK1 |
| Piceatannol | FGFR1 |
| Piceatannol | FOS |
| Piceatannol | GRK5 |
| Piceatannol | GSK3A |
| Piceatannol | GSK3B |
| Piceatannol | HCK |
| Piceatannol | IKKE |
| Piceatannol | JAK2 |
| Piceatannol | JAK3 |
| Piceatannol | KAPCA |
| Piceatannol | KC1A |
| Piceatannol | KCC1D |
| Piceatannol | KCC2A |
| Piceatannol | KKCC2 |
| Piceatannol | KPCT |
| Piceatannol | KS6A3 |
| Piceatannol | LGUL |
| Piceatannol | LOX15 |
| Piceatannol | LOX5 |
| Piceatannol | LRRK2 |
| Piceatannol | LTK |
| Piceatannol | M3K10 |
| Piceatannol | M4K4 |
| Piceatannol | MARK3 |
| Piceatannol | MARK4 |
| Piceatannol | MELK |
| Piceatannol | MK10 |
| Piceatannol | MMP1 |
| Piceatannol | MMP9 |
| Piceatannol | NEMO |
| Piceatannol | NF2L2 |
| Piceatannol | NFKB1 |
| Piceatannol | NQO2 |
| Piceatannol | PGFRA |
| Piceatannol | PGH1 |
| Piceatannol | PIM1 |
| Piceatannol | PPBI |
| Piceatannol | PPBN |
| Piceatannol | PRKX |
| Piceatannol | RET |
| Piceatannol | ROCK1 |
| Piceatannol | ROCK2 |
| Piceatannol | ROS1 |
| Piceatannol | SGK2 |
| Piceatannol | SIK2 |
| Piceatannol | ST17B |
| Piceatannol | STK3 |
| Piceatannol | SYUA |
| Piceatannol | TAOK1 |
| Piceatannol | TAU |
| Piceatannol | TBB1 |
| Piceatannol | TBB3 |
| Piceatannol | TBK1 |
| Piceatannol | TF65 |
| Piceatannol | TTHY |
| Piceatannol | TYRO3 |
| Piceatannol | LCK |
| Piceatannol | KSYK |
| Piceatannol | PGH1 |
| Piceatannol | NQO2 |
| Piceatannol | ESR1 |
| Piceatannol | CAH7 |
| Piceatannol | CAH12 |
| Piceatannol | CAH4 |
| Piceatannol | AOFA |
| Piceatannol | CAH2 |
| Piceatannol | SC6A2 |
| Piceatannol | PGH2 |
| Piceatannol | A4 |
| Piceatannol | CAH1 |
| Piceatannol | CAH3 |
| Piceatannol | MMP2 |
| Piceatannol | MMP9 |
| Piceatannol | PLGF |
| Piceatannol | VEGFA |
| Piceatannol | BACE1 |
| Piceatannol | CAH9 |
| Piceatannol | ERG1 |
| Piceatannol | MMP13 |
| Piceatannol | MMP12 |
| Piceatannol | CAH6 |
| Piceatannol | CAH5B |
| Piceatannol | BMP2 |
| Piceatannol | ALDR |
| Piceatannol | THRB |
| Piceatannol | MAP2 |
| Piceatannol | PPP5 |
| Piceatannol | PTN1 |
| Piceatannol | ALBU |
| Piceatannol | CAH2 |
| Piceatannol | DUS6 |
| Piceatannol | PIM1 |
| Piceatannol | PPARG |
| Piceatannol | AK1C2 |
| Piceatannol | CHLE |
| Piceatannol | CCNA2 |
| Piceatannol | MK14 |
| Piceatannol | CTNA1 |
| Piceatannol | PLGF |
| Piceatannol | TTHY |
| Piceatannol | GLRX1 |
| Piceatannol | VGFR1 |
| Oxyresveratrol | A4 |
| Oxyresveratrol | MK01 |
| Oxyresveratrol | ACHA7 |
| Oxyresveratrol | CAH3 |
| Oxyresveratrol | CAH5A |
| Oxyresveratrol | CAH5B |
| Oxyresveratrol | CAH6 |
| Oxyresveratrol | CP1A1 |
| Oxyresveratrol | CP1B1 |
| Oxyresveratrol | ECE2 |
| Oxyresveratrol | KKCC2 |
| Oxyresveratrol | LGUL |
| Oxyresveratrol | LOX5 |
| Oxyresveratrol | MARK4 |
| Oxyresveratrol | NF2L2 |
| Oxyresveratrol | NQO2 |
| Oxyresveratrol | PGH1 |
| Oxyresveratrol | TAU |
| Oxyresveratrol | TBB1 |
| Oxyresveratrol | TBB3 |
| Oxyresveratrol | TTHY |
| Oxyresveratrol | PGH1 |
| Oxyresveratrol | LCK |
| Oxyresveratrol | KSYK |
| Oxyresveratrol | NQO2 |
| Oxyresveratrol | ESR1 |
| Oxyresveratrol | AOFA |
| Oxyresveratrol | CAH2 |
| Oxyresveratrol | SC6A2 |
| Oxyresveratrol | PGH2 |
| Oxyresveratrol | CAH7 |
| Oxyresveratrol | A4 |
| Oxyresveratrol | CAH1 |
| Oxyresveratrol | CAH3 |
| Oxyresveratrol | CAH6 |
| Oxyresveratrol | PK3CB |
| Oxyresveratrol | STS |
| Oxyresveratrol | APOA2 |
| Oxyresveratrol | ALDR |
| Oxyresveratrol | ALBU |
| Oxyresveratrol | THRB |
| Oxyresveratrol | KIF11 |
| Oxyresveratrol | BACE1 |
| Oxyresveratrol | PTN1 |
| Oxyresveratrol | CAH2 |
| Oxyresveratrol | PIM1 |
| Oxyresveratrol | MAP2 |
| Oxyresveratrol | TTHY |
| Oxyresveratrol | PPARG |
| Oxyresveratrol | VTDB |
| Oxyresveratrol | SRC |
| Oxyresveratrol | ADA17 |
| Oxyresveratrol | GLRX1 |
| Oxyresveratrol | KAPCA_BOVIN |
| Oxyresveratrol | ADHX |
| Oxyresveratrol | TYRO |
| Butyl β-D-fructopyranoside | GBA2 |
| Butyl β-D-fructopyranoside | GDE |
| Butyl β-D-fructopyranoside | GLCM |
| Butyl β-D-fructopyranoside | LYAG |
| Butyl β-D-fructopyranoside | MGA |
| Butyl β-D-fructopyranoside | HS90A |
| Butyl β-D-fructopyranoside | VEGFA |
| Butyl β-D-fructopyranoside | PSN2 |
| Butyl β-D-fructopyranoside | PEN2 |
| Butyl β-D-fructopyranoside | NICA |
| Butyl β-D-fructopyranoside | APH1A |
| Butyl β-D-fructopyranoside | PSN1 |
| Butyl β-D-fructopyranoside | APH1B |
| Butyl β-D-fructopyranoside | FGF1 |
| Butyl β-D-fructopyranoside | FGF2 |
| Butyl β-D-fructopyranoside | HPSE |
| Butyl β-D-fructopyranoside | PPIA |
| Butyl β-D-fructopyranoside | PDE5A |
| Butyl β-D-fructopyranoside | CAH2 |
| Butyl β-D-fructopyranoside | THRB |
| Butyl β-D-fructopyranoside | KINH |
| Butyl β-D-fructopyranoside | NR1H2 |
| Butyl β-D-fructopyranoside | PTN1 |
| Butyl β-D-fructopyranoside | CATB |
| Butyl β-D-fructopyranoside | SAMP |
| Butyl β-D-fructopyranoside | MAOM |
| Butyl β-D-fructopyranoside | CDK2 |
| Butyl β-D-fructopyranoside | CHLE |
| Butyl β-D-fructopyranoside | ALBU |
| Butyl β-D-fructopyranoside | AK1C1 |
| Butyl β-D-fructopyranoside | SRC |
| Butyl β-D-fructopyranoside | BCAT2 |
| Butyl β-D-fructopyranoside | PPARG |
| Butyl β-D-fructopyranoside | LV208 |
| Butyl β-D-fructopyranoside | FGF1 |
| Butyl β-D-fructopyranoside | SUIS |
| Syringic acid | A4 |
| Syringic acid | ABCG2 |
| Syringic acid | AK1C1 |
| Syringic acid | AK1C2 |
| Syringic acid | AK1C3 |
| Syringic acid | ALR |
| Syringic acid | CAH13 |
| Syringic acid | CAH12 |
| Syringic acid | CAH14 |
| Syringic acid | CAH3 |
| Syringic acid | CAH4 |
| Syringic acid | CAH1 |
| Syringic acid | CAH2 |
| Syringic acid | CAH5A |
| Syringic acid | CAH5B |
| Syringic acid | CAH6 |
| Syringic acid | CAH7 |
| Syringic acid | CAH9 |
| Syringic acid | CP26B |
| Syringic acid | DHPR |
| Syringic acid | DPOLA |
| Syringic acid | DPOLB |
| Syringic acid | DUS3 |
| Syringic acid | DYN1 |
| Syringic acid | ERN1 |
| Syringic acid | EST1 |
| Syringic acid | EST2 |
| Syringic acid | FOS |
| Syringic acid | FTO |
| Syringic acid | FUT7 |
| Syringic acid | G6PT1 |
| Syringic acid | GBRB1 |
| Syringic acid | GRK6 |
| Syringic acid | HCAR3 |
| Syringic acid | HIF1N |
| Syringic acid | HMDH |
| Syringic acid | HNF4A |
| Syringic acid | IBP3 |
| Syringic acid | JUN |
| Syringic acid | KCNK9 |
| Syringic acid | KDM2A |
| Syringic acid | KDM2B |
| Syringic acid | KDM3A |
| Syringic acid | KDM4C |
| Syringic acid | KDM4D |
| Syringic acid | KDM4E |
| Syringic acid | KDM5A |
| Syringic acid | KDM5B |
| Syringic acid | KDM5C |
| Syringic acid | KLF5 |
| Syringic acid | KLK5 |
| Syringic acid | KMO |
| Syringic acid | LDHB |
| Syringic acid | LGUL |
| Syringic acid | LPAR1 |
| Syringic acid | LPAR5 |
| Syringic acid | LT4R1 |
| Syringic acid | MDHC |
| Syringic acid | MDR1 |
| Syringic acid | NF2L2 |
| Syringic acid | NFKB1 |
| Syringic acid | OXDD |
| Syringic acid | P3C2G |
| Syringic acid | P4HA1 |
| Syringic acid | P4HTM |
| Syringic acid | PA24A |
| Syringic acid | PA24B |
| Syringic acid | PA2GD |
| Syringic acid | PE2R1 |
| Syringic acid | PI3R5 |
| Syringic acid | PTN12 |
| Syringic acid | PTN5 |
| Syringic acid | PTN6 |
| Syringic acid | PTN7 |
| Syringic acid | RARA |
| Syringic acid | RARB |
| Syringic acid | RARG |
| Syringic acid | RNH1 |
| Syringic acid | S13A5 |
| Syringic acid | S5A2 |
| Syringic acid | SAE1 |
| Syringic acid | SAE2 |
| Syringic acid | SIR5 |
| Syringic acid | T2R14 |
| Syringic acid | TAU |
| Syringic acid | TBB1 |
| Syringic acid | TNR1A |
| Syringic acid | TPMT |
| Syringic acid | CAH2 |
| Syringic acid | CAH7 |
| Syringic acid | CAH1 |
| Syringic acid | CAH3 |
| Syringic acid | CAH6 |
| Syringic acid | CAH12 |
| Syringic acid | CAH14 |
| Syringic acid | CAH9 |
| Syringic acid | PDE5A |
| Syringic acid | SNRPA |
| Syringic acid | ANGI |
| Syringic acid | CHLE |
| Syringic acid | BCAT2 |
| Syringic acid | DDX6 |
| Syringic acid | B3GA1 |
| Syringic acid | SRC |
| Syringic acid | PNPH |
| Syringic acid | PCKGC |
| Syringic acid | CBPB1 |
| Syringic acid | ECP |
| Syringic acid | HS90A |
| Syringic acid | PTN1 |
| Syringic acid | RNAS4 |
| Syringic acid | CDK6 |
| Syringic acid | LEG2 |
| Syringic acid | CP2C9 |
| Syringic acid | SODM |
| Syringic acid | MK14 |
| Syringic acid | CAH5A |
| Syringic acid | TS1R1 |
| Syringic acid | TTHY |
| Protocatechuic acid | 3MG |
| Protocatechuic acid | AK1C2 |
| Protocatechuic acid | AK1C3 |
| Protocatechuic acid | AK1C1 |
| Protocatechuic acid | AK1C4 |
| Protocatechuic acid | ALR |
| Protocatechuic acid | AMPE |
| Protocatechuic acid | APEX1 |
| Protocatechuic acid | BHMT1 |
| Protocatechuic acid | CAH14 |
| Protocatechuic acid | CAH12 |
| Protocatechuic acid | CAH3 |
| Protocatechuic acid | CAH1 |
| Protocatechuic acid | CAH4 |
| Protocatechuic acid | CAH2 |
| Protocatechuic acid | CAH5A |
| Protocatechuic acid | CAH5B |
| Protocatechuic acid | CAH6 |
| Protocatechuic acid | CAH7 |
| Protocatechuic acid | CAH9 |
| Protocatechuic acid | CH60 |
| Protocatechuic acid | CP26B |
| Protocatechuic acid | DHB14 |
| Protocatechuic acid | DHB1 |
| Protocatechuic acid | DHPR |
| Protocatechuic acid | DOPO |
| Protocatechuic acid | DPOLB |
| Protocatechuic acid | DYN1 |
| Protocatechuic acid | ERCC1 |
| Protocatechuic acid | ERN1 |
| Protocatechuic acid | EST1 |
| Protocatechuic acid | EST2 |
| Protocatechuic acid | FOLH1 |
| Protocatechuic acid | FTO |
| Protocatechuic acid | FUT7 |
| Protocatechuic acid | FYN |
| Protocatechuic acid | GABT |
| Protocatechuic acid | GSTA1 |
| Protocatechuic acid | HCAR3 |
| Protocatechuic acid | HIF1N |
| Protocatechuic acid | IBP1 |
| Protocatechuic acid | IBP2 |
| Protocatechuic acid | IBP3 |
| Protocatechuic acid | IBP4 |
| Protocatechuic acid | IBP5 |
| Protocatechuic acid | IBP6 |
| Protocatechuic acid | KCMA1 |
| Protocatechuic acid | KDM2A |
| Protocatechuic acid | KDM2B |
| Protocatechuic acid | KDM3A |
| Protocatechuic acid | KDM4A |
| Protocatechuic acid | KDM4C |
| Protocatechuic acid | KDM4D |
| Protocatechuic acid | KDM4E |
| Protocatechuic acid | KDM5B |
| Protocatechuic acid | KDM5A |
| Protocatechuic acid | KDM5C |
| Protocatechuic acid | KDM6B |
| Protocatechuic acid | KKCC2 |
| Protocatechuic acid | KMO |
| Protocatechuic acid | KS6B2 |
| Protocatechuic acid | LOX15 |
| Protocatechuic acid | MPIP2 |
| Protocatechuic acid | NEUR3 |
| Protocatechuic acid | NR1H4 |
| Protocatechuic acid | NR4A1 |
| Protocatechuic acid | NR4A2 |
| Protocatechuic acid | NSD2 |
| Protocatechuic acid | OXDD |
| Protocatechuic acid | P4HA1 |
| Protocatechuic acid | P4HTM |
| Protocatechuic acid | PA24B |
| Protocatechuic acid | PA2GD |
| Protocatechuic acid | PAI1 |
| Protocatechuic acid | PLAP |
| Protocatechuic acid | PLEC |
| Protocatechuic acid | POLH |
| Protocatechuic acid | POLI |
| Protocatechuic acid | PPBN |
| Protocatechuic acid | PPBT |
| Protocatechuic acid | RARA |
| Protocatechuic acid | RARB |
| Protocatechuic acid | RARG |
| Protocatechuic acid | RHOA |
| Protocatechuic acid | RXRA |
| Protocatechuic acid | RXRB |
| Protocatechuic acid | RXRG |
| Protocatechuic acid | S22A6 |
| Protocatechuic acid | S5A2 |
| Protocatechuic acid | SSDH |
| Protocatechuic acid | SYUA |
| Protocatechuic acid | TOP1 |
| Protocatechuic acid | TPMT |
| Protocatechuic acid | TTHY |
| Protocatechuic acid | TY3H |
| Protocatechuic acid | XCT |
| Protocatechuic acid | CAH2 |
| Protocatechuic acid | CAH7 |
| Protocatechuic acid | CAH1 |
| Protocatechuic acid | CAH6 |
| Protocatechuic acid | CAH12 |
| Protocatechuic acid | CAH14 |
| Protocatechuic acid | CAH9 |
| Protocatechuic acid | ODPB |
| Protocatechuic acid | PTN1 |
| Protocatechuic acid | CBPB1 |
| Protocatechuic acid | PCKGC |
| Protocatechuic acid | RAN |
| Protocatechuic acid | SRC |
| Protocatechuic acid | PIPNA |
| Protocatechuic acid | ST1E1 |
| Protocatechuic acid | FOLH1 |
| Protocatechuic acid | RB11A |
| Protocatechuic acid | DYR |
| Protocatechuic acid | PMS2 |
| Protocatechuic acid | FPPS |
| Protocatechuic acid | KIF11 |
| Protocatechuic acid | RAB9A |
| Protocatechuic acid | NMNA3 |
| Protocatechuic acid | UCK2 |
| Protocatechuic acid | RNAS2 |
| Protocatechuic acid | RND3 |
| Protocatechuic acid | GSK3B |
| Protocatechuic acid | CAH4 |
| Protocatechuic acid | XPF |
| Vanillic acid | 3MG |
| Vanillic acid | A4 |
| Vanillic acid | ABCG2 |
| Vanillic acid | ACES |
| Vanillic acid | ACPM |
| Vanillic acid | AK1C2 |
| Vanillic acid | AK1C3 |
| Vanillic acid | ALR |
| Vanillic acid | AOFA |
| Vanillic acid | AOFB |
| Vanillic acid | APEX1 |
| Vanillic acid | ATG4B |
| Vanillic acid | BGLR |
| Vanillic acid | BRAF |
| Vanillic acid | BRSK1 |
| Vanillic acid | CAH13 |
| Vanillic acid | CAH12 |
| Vanillic acid | CAH14 |
| Vanillic acid | CAH3 |
| Vanillic acid | CAH4 |
| Vanillic acid | CAH1 |
| Vanillic acid | CAH2 |
| Vanillic acid | CAH5B |
| Vanillic acid | CAH7 |
| Vanillic acid | CAH6 |
| Vanillic acid | CAH5A |
| Vanillic acid | CAH9 |
| Vanillic acid | CBP |
| Vanillic acid | CCND3 |
| Vanillic acid | CHK1 |
| Vanillic acid | CIA30 |
| Vanillic acid | CISD1 |
| Vanillic acid | CP1B1 |
| Vanillic acid | CP26A |
| Vanillic acid | CP26B |
| Vanillic acid | CPT1A |
| Vanillic acid | CPT2 |
| Vanillic acid | CPT1B |
| Vanillic acid | CSK21 |
| Vanillic acid | CSK22 |
| Vanillic acid | CTDS1 |
| Vanillic acid | DAPK3 |
| Vanillic acid | DCOR |
| Vanillic acid | DEFM |
| Vanillic acid | DHCR7 |
| Vanillic acid | DHPR |
| Vanillic acid | DPOLB |
| Vanillic acid | DRD1 |
| Vanillic acid | DUS3 |
| Vanillic acid | DYR1A |
| Vanillic acid | DYRK3 |
| Vanillic acid | DYRK4 |
| Vanillic acid | EBP |
| Vanillic acid | EGFR |
| Vanillic acid | EGLN1 |
| Vanillic acid | EP300 |
| Vanillic acid | ERN1 |
| Vanillic acid | EST1 |
| Vanillic acid | EST2 |
| Vanillic acid | FAK1 |
| Vanillic acid | FOLH1 |
| Vanillic acid | FOS |
| Vanillic acid | FTO |
| Vanillic acid | FUT7 |
| Vanillic acid | FYN |
| Vanillic acid | G6PT1 |
| Vanillic acid | GLR |
| Vanillic acid | HCAR3 |
| Vanillic acid | HDAC8 |
| Vanillic acid | HIF1N |
| Vanillic acid | HNF4A |
| Vanillic acid | HPSE |
| Vanillic acid | IBP1 |
| Vanillic acid | IBP2 |
| Vanillic acid | IBP3 |
| Vanillic acid | IBP4 |
| Vanillic acid | IBP5 |
| Vanillic acid | IBP6 |
| Vanillic acid | IF4H |
| Vanillic acid | JUN |
| Vanillic acid | KC1G2 |
| Vanillic acid | KC1G3 |
| Vanillic acid | KCC2A |
| Vanillic acid | KCC2B |
| Vanillic acid | KCC2D |
| Vanillic acid | KCC2G |
| Vanillic acid | KCMA1 |
| Vanillic acid | KCNB1 |
| Vanillic acid | KCNK9 |
| Vanillic acid | KDM2A |
| Vanillic acid | KDM2B |
| Vanillic acid | KDM4A |
| Vanillic acid | KDM3A |
| Vanillic acid | KDM4E |
| Vanillic acid | KDM4C |
| Vanillic acid | KDM4D |
| Vanillic acid | KDM5A |
| Vanillic acid | KDM5B |
| Vanillic acid | KDM5C |
| Vanillic acid | KDM6B |
| Vanillic acid | KLF5 |
| Vanillic acid | KLK5 |
| Vanillic acid | KLK7 |
| Vanillic acid | KMO |
| Vanillic acid | KS6B2 |
| Vanillic acid | LGUL |
| Vanillic acid | LOX5 |
| Vanillic acid | LPAR1 |
| Vanillic acid | LPAR5 |
| Vanillic acid | LRRK2 |
| Vanillic acid | LT4R1 |
| Vanillic acid | MAX |
| Vanillic acid | MCL1 |
| Vanillic acid | MDHC |
| Vanillic acid | MDR1 |
| Vanillic acid | MIF |
| Vanillic acid | MITF |
| Vanillic acid | MMP15 |
| Vanillic acid | MMP16 |
| Vanillic acid | MMP26 |
| Vanillic acid | MOT4 |
| Vanillic acid | MPRI |
| Vanillic acid | MYC |
| Vanillic acid | NDUA1 |
| Vanillic acid | NDUA2 |
| Vanillic acid | NDUA3 |
| Vanillic acid | NDUA4 |
| Vanillic acid | NDUA5 |
| Vanillic acid | NDUA6 |
| Vanillic acid | NDUA8 |
| Vanillic acid | NDUA7 |
| Vanillic acid | NDUA9 |
| Vanillic acid | NDUAA |
| Vanillic acid | NDUAB |
| Vanillic acid | NDUAC |
| Vanillic acid | NDUAD |
| Vanillic acid | NDUB1 |
| Vanillic acid | NDUB2 |
| Vanillic acid | NDUB3 |
| Vanillic acid | NDUB4 |
| Vanillic acid | NDUB5 |
| Vanillic acid | NDUB6 |
| Vanillic acid | NDUB7 |
| Vanillic acid | NDUB8 |
| Vanillic acid | NDUB9 |
| Vanillic acid | NDUBA |
| Vanillic acid | NDUBB |
| Vanillic acid | NDUC1 |
| Vanillic acid | NDUC2 |
| Vanillic acid | NDUF2 |
| Vanillic acid | NDUF3 |
| Vanillic acid | NDUF4 |
| Vanillic acid | NDUS1 |
| Vanillic acid | NDUS2 |
| Vanillic acid | NDUS3 |
| Vanillic acid | NDUS4 |
| Vanillic acid | NDUS5 |
| Vanillic acid | NDUS6 |
| Vanillic acid | NDUS7 |
| Vanillic acid | NDUS8 |
| Vanillic acid | NDUV1 |
| Vanillic acid | NDUV2 |
| Vanillic acid | NDUV3 |
| Vanillic acid | NEMO |
| Vanillic acid | NEUR3 |
| Vanillic acid | NF2L2 |
| Vanillic acid | NFKB1 |
| Vanillic acid | NOX4 |
| Vanillic acid | NR1H4 |
| Vanillic acid | NR4A1 |
| Vanillic acid | NR4A2 |
| Vanillic acid | NSD2 |
| Vanillic acid | NU2M |
| Vanillic acid | NU1M |
| Vanillic acid | NU3M |
| Vanillic acid | NU4LM |
| Vanillic acid | NU4M |
| Vanillic acid | NU5M |
| Vanillic acid | NU6M |
| Vanillic acid | NUA4L |
| Vanillic acid | P3C2G |
| Vanillic acid | P4HA1 |
| Vanillic acid | P4HTM |
| Vanillic acid | PA24A |
| Vanillic acid | PA24B |
| Vanillic acid | PA2GD |
| Vanillic acid | PACR |
| Vanillic acid | PAX8 |
| Vanillic acid | PDE4C |
| Vanillic acid | PDE4B |
| Vanillic acid | PDE4D |
| Vanillic acid | PDK3 |
| Vanillic acid | PDK4 |
| Vanillic acid | PE2R1 |
| Vanillic acid | PERM |
| Vanillic acid | PGFRA |
| Vanillic acid | PHKG2 |
| Vanillic acid | PLAP |
| Vanillic acid | POLH |
| Vanillic acid | POLI |
| Vanillic acid | PPBN |
| Vanillic acid | PPBT |
| Vanillic acid | PTN12 |
| Vanillic acid | PTN1 |
| Vanillic acid | PTN6 |
| Vanillic acid | PTN7 |
| Vanillic acid | PTPRC |
| Vanillic acid | PYGL |
| Vanillic acid | RAF1 |
| Vanillic acid | RARA |
| Vanillic acid | RARB |
| Vanillic acid | RARG |
| Vanillic acid | RBBP9 |
| Vanillic acid | RET |
| Vanillic acid | RHOA |
| Vanillic acid | RNH1 |
| Vanillic acid | ROCK1 |
| Vanillic acid | ROCK2 |
| Vanillic acid | RXRA |
| Vanillic acid | RXRB |
| Vanillic acid | RXRG |
| Vanillic acid | S13A5 |
| Vanillic acid | S22A3 |
| Vanillic acid | S22A6 |
| Vanillic acid | S5A2 |
| Vanillic acid | SAE1 |
| Vanillic acid | SAE2 |
| Vanillic acid | SC5A7 |
| Vanillic acid | SC6A5 |
| Vanillic acid | SENP7 |
| Vanillic acid | SHBG |
| Vanillic acid | SIR5 |
| Vanillic acid | SRPK1 |
| Vanillic acid | ST17A |
| Vanillic acid | ST17B |
| Vanillic acid | STS |
| Vanillic acid | T2R14 |
| Vanillic acid | TAAR1 |
| Vanillic acid | TAOK1 |
| Vanillic acid | TAU |
| Vanillic acid | TBB1 |
| Vanillic acid | TBB3 |
| Vanillic acid | TF |
| Vanillic acid | THA |
| Vanillic acid | THB |
| Vanillic acid | TNR1A |
| Vanillic acid | TOPK |
| Vanillic acid | TPMT |
| Vanillic acid | TRPM8 |
| Vanillic acid | TRPV1 |
| Vanillic acid | TS1R1 |
| Vanillic acid | TSSK2 |
| Vanillic acid | TTHY |
| Vanillic acid | CAH2 |
| Vanillic acid | CAH7 |
| Vanillic acid | CAH1 |
| Vanillic acid | CAH12 |
| Vanillic acid | CAH14 |
| Vanillic acid | CAH9 |
| Vanillic acid | CAH3 |
| Vanillic acid | CAH6 |
| Vanillic acid | CAH5A |
| Vanillic acid | CAH4 |
| Vanillic acid | TPMT |
| Vanillic acid | TTHY |
| Vanillic acid | AK1C3 |
| Vanillic acid | CAH5B |
| Vanillic acid | CAH13 |
| Vanillic acid | PDE5A |
| Vanillic acid | ANGI |
| Vanillic acid | CHLE |
| Vanillic acid | DDX6 |
| Vanillic acid | B3GA1 |
| Vanillic acid | CBPB1 |
| Vanillic acid | HS90A |
| Vanillic acid | PCKGC |
| Vanillic acid | PTN1 |
| Vanillic acid | CDK6 |
| Vanillic acid | MK14 |
| Vanillic acid | SODM |
| Vanillic acid | ECP |
| Vanillic acid | SRC |
| Vanillic acid | PYRD |
| Vanillic acid | HMDH |
| Vanillic acid | TPIS |
| Vanillic acid | INSR |
| Vanillic acid | ISG20 |
| Vanillic acid | CDK2 |
| Vanillic acid | XCT |
| 3,5-Dimethoxy-4-β-D-glucopyranosylcinnamic acid | AL1A2 |
| 3,5-Dimethoxy-4-β-D-glucopyranosylcinnamic acid | AL1B1 |
| 3,5-Dimethoxy-4-β-D-glucopyranosylcinnamic acid | AMYP |
| 3,5-Dimethoxy-4-β-D-glucopyranosylcinnamic acid | B4GT1 |
| 3,5-Dimethoxy-4-β-D-glucopyranosylcinnamic acid | BGAL |
| 3,5-Dimethoxy-4-β-D-glucopyranosylcinnamic acid | CAH12 |
| 3,5-Dimethoxy-4-β-D-glucopyranosylcinnamic acid | CAH1 |
| 3,5-Dimethoxy-4-β-D-glucopyranosylcinnamic acid | CAH9 |
| 3,5-Dimethoxy-4-β-D-glucopyranosylcinnamic acid | CALM1 |
| 3,5-Dimethoxy-4-β-D-glucopyranosylcinnamic acid | CD69 |
| 3,5-Dimethoxy-4-β-D-glucopyranosylcinnamic acid | ERAP1 |
| 3,5-Dimethoxy-4-β-D-glucopyranosylcinnamic acid | FGF1 |
| 3,5-Dimethoxy-4-β-D-glucopyranosylcinnamic acid | FGF2 |
| 3,5-Dimethoxy-4-β-D-glucopyranosylcinnamic acid | G3P |
| 3,5-Dimethoxy-4-β-D-glucopyranosylcinnamic acid | IL2 |
| 3,5-Dimethoxy-4-β-D-glucopyranosylcinnamic acid | IL6 |
| 3,5-Dimethoxy-4-β-D-glucopyranosylcinnamic acid | LEG1 |
| 3,5-Dimethoxy-4-β-D-glucopyranosylcinnamic acid | LEG3 |
| 3,5-Dimethoxy-4-β-D-glucopyranosylcinnamic acid | LEG4 |
| 3,5-Dimethoxy-4-β-D-glucopyranosylcinnamic acid | LEG8 |
| 3,5-Dimethoxy-4-β-D-glucopyranosylcinnamic acid | LEG7 |
| 3,5-Dimethoxy-4-β-D-glucopyranosylcinnamic acid | LEG9 |
| 3,5-Dimethoxy-4-β-D-glucopyranosylcinnamic acid | LYAM1 |
| 3,5-Dimethoxy-4-β-D-glucopyranosylcinnamic acid | LYAM3 |
| 3,5-Dimethoxy-4-β-D-glucopyranosylcinnamic acid | PDIA1 |
| 3,5-Dimethoxy-4-β-D-glucopyranosylcinnamic acid | PSA |
| 3,5-Dimethoxy-4-β-D-glucopyranosylcinnamic acid | S28A3 |
| 3,5-Dimethoxy-4-β-D-glucopyranosylcinnamic acid | SC5A1 |
| 3,5-Dimethoxy-4-β-D-glucopyranosylcinnamic acid | SC5A2 |
| 3,5-Dimethoxy-4-β-D-glucopyranosylcinnamic acid | SC5A4 |
| 3,5-Dimethoxy-4-β-D-glucopyranosylcinnamic acid | SC5AB |
| 3,5-Dimethoxy-4-β-D-glucopyranosylcinnamic acid | TYDP1 |
| 3,5-Dimethoxy-4-β-D-glucopyranosylcinnamic acid | TYRO |
| 3,5-Dimethoxy-4-β-D-glucopyranosylcinnamic acid | GSTP1 |
| 3,5-Dimethoxy-4-β-D-glucopyranosylcinnamic acid | CMA1 |
| 3,5-Dimethoxy-4-β-D-glucopyranosylcinnamic acid | DDX6 |
| 3,5-Dimethoxy-4-β-D-glucopyranosylcinnamic acid | GLCM |
| 3,5-Dimethoxy-4-β-D-glucopyranosylcinnamic acid | PPIA |
| 3,5-Dimethoxy-4-β-D-glucopyranosylcinnamic acid | NR1H2 |
| 3,5-Dimethoxy-4-β-D-glucopyranosylcinnamic acid | PNPH |
| 3,5-Dimethoxy-4-β-D-glucopyranosylcinnamic acid | CAH2 |
| 3,5-Dimethoxy-4-β-D-glucopyranosylcinnamic acid | ALDR |
| 3,5-Dimethoxy-4-β-D-glucopyranosylcinnamic acid | CATD |
| 3,5-Dimethoxy-4-β-D-glucopyranosylcinnamic acid | ANGI |
| 3,5-Dimethoxy-4-β-D-glucopyranosylcinnamic acid | PDE4B |
| 3,5-Dimethoxy-4-β-D-glucopyranosylcinnamic acid | CDK2 |
| 3,5-Dimethoxy-4-β-D-glucopyranosylcinnamic acid | MAOM |
| 3,5-Dimethoxy-4-β-D-glucopyranosylcinnamic acid | C1TC |
| 3,5-Dimethoxy-4-β-D-glucopyranosylcinnamic acid | CSK21 |
| 3,5-Dimethoxy-4-β-D-glucopyranosylcinnamic acid | PH4H |
| 3,5-Dimethoxy-4-β-D-glucopyranosylcinnamic acid | PIM1 |
| 3,5-Dimethoxy-4-β-D-glucopyranosylcinnamic acid | KINH |
| 3,5-Dimethoxy-4-β-D-glucopyranosylcinnamic acid | MIF |
| 3,5-Dimethoxy-4-β-D-glucopyranosylcinnamic acid | VEGFA |
| Maackoline | TYSY |
| Maackoline | ALDR |
| Maackoline | BACE1 |
| Maackoline | PTN1 |
| Maackoline | CDK1 |
| Maackoline | SCN9A |
| Maackoline | CHK1 |
| Maackoline | AK1C2 |
| Maackoline | BMP2 |
| Maackoline | CASP7 |
| Maackoline | PIM1 |
| Maackoline | MAPK2 |
| Maackoline | ALBU |
| Maackoline | EPHB4 |
| Maackoline | CAH2 |
| Maackoline | CASP3 |
| Maackoline | GSTP1 |
| Maackoline | PK3CG |
| Maackoline | ALDR |
| Maackoline | ANDR |
| Maackoline | THB |
| Maackoline | CP19A |
| Maackoline | PTN1 |
| Maackoline | VTDB |
| Maackoline | THRB |
| Maackoline | PNPH |
| Maackoline | TYSY |
| Maackoline | PRGR |
| Maackoline | PDE5A |
| Maackoline | PDE4B |
| Maackoline | MK01 |
| Maackoline | RORA |
| Maackoline | AK1C3 |
| Maackoline | STS |
| Maackoline | ESR1 |
| Maackoline | EGFR |
| Maackoline | ERR3 |
| Maackoline | NGAL |
| Maackoline | SNRPA |
| Maackoline | ANGI |
| Maackoline | TGFR1 |
| Maackoline | WEE1 |
| Moracin M | PGH1 |
| Moracin M | MK01 |
| Moracin M | PDE4B |
| Moracin M | PDE4D |
| Moracin M | SIR1 |
| Moracin M | CD38 |
| Moracin M | SHBG |
| Moracin M | ESR1 |
| Moracin M | PK3CG |
| Moracin M | ANDR |
| Moracin M | PPARG |
| Moracin M | CAH2 |
| Moracin M | THRB |
| Moracin M | PDE4D |
| Moracin M | PIM1 |
| Moracin M | PA2GX |
| Moracin M | CCNA2 |
| Moracin M | PTN1 |
| Moracin M | EST1 |
| Moracin M | GSK3B |
| Moracin M | AK1C1 |
| Moracin M | MAP2 |
| Moracin M | CTNA1 |
| Moracin M | SHBG |
| Moracin M | HPGDS |
| Moracin M | DPP4 |
| Moracin M | ESR2 |
| Gallic acid | PTN1 |
| Gallic acid | ODPB |
| Gallic acid | CBPB1 |
| Gallic acid | PCKGC |
| Gallic acid | SRC |
| Gallic acid | CDK7 |
| Gallic acid | PIPNA |
| Gallic acid | MK10 |
| Gallic acid | FOLH1 |
| Gallic acid | ST1E1 |
| Gallic acid | PMS2 |
| Gallic acid | DYR |
| Gallic acid | MK12 |
| Gallic acid | KIF11 |
| Gallic acid | RB11A |
| Gallic acid | F261_RAT |
| Gallic acid | NMNA3 |
| Gallic acid | FPPS |
| Gallic acid | RNAS2 |
| Gallic acid | CAH2 |
| Gallic acid | CAH7 |
| Gallic acid | CAH1 |
| Gallic acid | CAH3 |
| Gallic acid | CAH6 |
| Gallic acid | CAH12 |
| Gallic acid | CAH14 |
| Gallic acid | CAH9 |
| Gallic acid | FUT7 |
| Gallic acid | CAH4 |
| Gallic acid | CAH5B |
| Gallic acid | CAH5A |
| Gallic acid | CAH13 |
| Gallic acid | RND3 |
| Trans-2-hydroxycinnamic acid | ABCG2 |
| Trans-2-hydroxycinnamic acid | ACHA7 |
| Trans-2-hydroxycinnamic acid | AK1BA |
| Trans-2-hydroxycinnamic acid | AK1C3 |
| Trans-2-hydroxycinnamic acid | AK1C4 |
| Trans-2-hydroxycinnamic acid | ALDR |
| Trans-2-hydroxycinnamic acid | AMD |
| Trans-2-hydroxycinnamic acid | AOFB |
| Trans-2-hydroxycinnamic acid | ARY1 |
| Trans-2-hydroxycinnamic acid | CAH14 |
| Trans-2-hydroxycinnamic acid | CAH12 |
| Trans-2-hydroxycinnamic acid | CAH3 |
| Trans-2-hydroxycinnamic acid | CAH1 |
| Trans-2-hydroxycinnamic acid | CAH2 |
| Trans-2-hydroxycinnamic acid | CAH5A |
| Trans-2-hydroxycinnamic acid | CAH5B |
| Trans-2-hydroxycinnamic acid | CAH6 |
| Trans-2-hydroxycinnamic acid | CAH7 |
| Trans-2-hydroxycinnamic acid | CP1A1 |
| Trans-2-hydroxycinnamic acid | CP26B |
| Trans-2-hydroxycinnamic acid | DOPO |
| Trans-2-hydroxycinnamic acid | DPYD |
| Trans-2-hydroxycinnamic acid | ECE2 |
| Trans-2-hydroxycinnamic acid | ERN1 |
| Trans-2-hydroxycinnamic acid | ESR1 |
| Trans-2-hydroxycinnamic acid | EST1 |
| Trans-2-hydroxycinnamic acid | EST2 |
| Trans-2-hydroxycinnamic acid | GBB1 |
| Trans-2-hydroxycinnamic acid | GBG2 |
| Trans-2-hydroxycinnamic acid | GBRB1 |
| Trans-2-hydroxycinnamic acid | GP183 |
| Trans-2-hydroxycinnamic acid | GRIK1 |
| Trans-2-hydroxycinnamic acid | GRIK2 |
| Trans-2-hydroxycinnamic acid | GRIK3 |
| Trans-2-hydroxycinnamic acid | GSTA1 |
| Trans-2-hydroxycinnamic acid | HS71A |
| Trans-2-hydroxycinnamic acid | LPAR2 |
| Trans-2-hydroxycinnamic acid | LPAR3 |
| Trans-2-hydroxycinnamic acid | MMP1 |
| Trans-2-hydroxycinnamic acid | MMP9 |
| Trans-2-hydroxycinnamic acid | NF2L2 |
| Trans-2-hydroxycinnamic acid | NFKB1 |
| Trans-2-hydroxycinnamic acid | NR0B2 |
| Trans-2-hydroxycinnamic acid | PA2GD |
| Trans-2-hydroxycinnamic acid | PAR14 |
| Trans-2-hydroxycinnamic acid | PE2R2 |
| Trans-2-hydroxycinnamic acid | PE2R3 |
| Trans-2-hydroxycinnamic acid | PE2R4 |
| Trans-2-hydroxycinnamic acid | RHOA |
| Trans-2-hydroxycinnamic acid | SENP7 |
| Trans-2-hydroxycinnamic acid | TAB1 |
| Trans-2-hydroxycinnamic acid | TAU |
| Trans-2-hydroxycinnamic acid | CAH2 |
| Trans-2-hydroxycinnamic acid | CAH1 |
| Trans-2-hydroxycinnamic acid | ALDR |
| Trans-2-hydroxycinnamic acid | CAH7 |
| Trans-2-hydroxycinnamic acid | ESR2 |
| Trans-2-hydroxycinnamic acid | CAH3 |
| Trans-2-hydroxycinnamic acid | CAH6 |
| Trans-2-hydroxycinnamic acid | CAH12 |
| Trans-2-hydroxycinnamic acid | CAH14 |
| Trans-2-hydroxycinnamic acid | CAH9 |
| Trans-2-hydroxycinnamic acid | CAH4 |
| Trans-2-hydroxycinnamic acid | CAH5B |
| Trans-2-hydroxycinnamic acid | GSTP1 |
| Trans-2-hydroxycinnamic acid | SAMP |
| Trans-2-hydroxycinnamic acid | SRC |
| Trans-2-hydroxycinnamic acid | CAH2 |
| Trans-2-hydroxycinnamic acid | B3GA1 |
| Trans-2-hydroxycinnamic acid | PTN1 |
| Trans-2-hydroxycinnamic acid | PA2GA |
| Trans-2-hydroxycinnamic acid | CO8G |
| Trans-2-hydroxycinnamic acid | CBPB1 |
| Trans-2-hydroxycinnamic acid | CDD |
| Trans-2-hydroxycinnamic acid | PH4H |
| Trans-2-hydroxycinnamic acid | PCKGC |
| Trans-2-hydroxycinnamic acid | LCK |
| Trans-2-hydroxycinnamic acid | TYSY |
| Trans-2-hydroxycinnamic acid | ADHX |
| Trans-2-hydroxycinnamic acid | PPAP |
| Trans-2-hydroxycinnamic acid | KTHY |
| Trans-2-hydroxycinnamic acid | AK1C2 |
| Trans-2-hydroxycinnamic acid | HCK |
| Trans-2-hydroxycinnamic acid | CP2C9 |
| Trans-2-hydroxycinnamic acid | CAH5A |
| Trans-2-hydroxycinnamic acid | TTHY |
| Trans-2-hydroxycinnamic acid | UPAR |
| Dihydroquercetin | 6PGD |
| Dihydroquercetin | MK01 |
| Dihydroquercetin | CAH12 |
| Dihydroquercetin | CAH3 |
| Dihydroquercetin | CAH4 |
| Dihydroquercetin | CAH6 |
| Dihydroquercetin | CAH7 |
| Dihydroquercetin | CBR1 |
| Dihydroquercetin | CP1B1 |
| Dihydroquercetin | CP2C8 |
| Dihydroquercetin | CP3A4 |
| Dihydroquercetin | DHPR |
| Dihydroquercetin | ELAV1 |
| Dihydroquercetin | ESR1 |
| Dihydroquercetin | ESR2 |
| Dihydroquercetin | FUT4 |
| Dihydroquercetin | FUT7 |
| Dihydroquercetin | KLK2 |
| Dihydroquercetin | LGUL |
| Dihydroquercetin | MMP12 |
| Dihydroquercetin | MRP2 |
| Dihydroquercetin | NEK6 |
| Dihydroquercetin | PLGF |
| Dihydroquercetin | SIAT6 |
| Dihydroquercetin | T2R31 |
| Dihydroquercetin | THRB |
| Dihydroquercetin | PK3CG |
| Dihydroquercetin | PDE5A |
| Dihydroquercetin | PRDX5 |
| Dihydroquercetin | ANDR |
| Dihydroquercetin | SNRPA |
| Dihydroquercetin | PPARG |
| Dihydroquercetin | CAH2 |
| Dihydroquercetin | ALBU |
| Dihydroquercetin | CCNA2 |
| Dihydroquercetin | PDE4D |
| Dihydroquercetin | CDK2 |
| Dihydroquercetin | MAP2 |
| Dihydroquercetin | BCAT2 |
| Dihydroquercetin | PA2GX |
| Dihydroquercetin | ALDR |
| Dihydroquercetin | CFAD |
| Dihydroquercetin | FGF1 |
| Dihydroquercetin | PTN1 |
| Dihydroquercetin | VEGFA |
| Dihydroquercetin | XDH |
| Oleanolic acid | 3MG |
| Oleanolic acid | AK1C2 |
| Oleanolic acid | AK1C3 |
| Oleanolic acid | AK1C4 |
| Oleanolic acid | AK1C1 |
| Oleanolic acid | ALR |
| Oleanolic acid | AMPE |
| Oleanolic acid | APEX1 |
| Oleanolic acid | BHMT1 |
| Oleanolic acid | CAH12 |
| Oleanolic acid | CAH14 |
| Oleanolic acid | CAH1 |
| Oleanolic acid | CAH2 |
| Oleanolic acid | CAH3 |
| Oleanolic acid | CAH4 |
| Oleanolic acid | CAH5A |
| Oleanolic acid | CAH5B |
| Oleanolic acid | CAH6 |
| Oleanolic acid | CAH7 |
| Oleanolic acid | CAH9 |
| Oleanolic acid | CH60 |
| Oleanolic acid | CP26B |
| Oleanolic acid | DHB14 |
| Oleanolic acid | DHB1 |
| Oleanolic acid | DHPR |
| Oleanolic acid | DOPO |
| Oleanolic acid | DPOLB |
| Oleanolic acid | DYN1 |
| Oleanolic acid | ERCC1 |
| Oleanolic acid | ERN1 |
| Oleanolic acid | EST1 |
| Oleanolic acid | EST2 |
| Oleanolic acid | FOLH1 |
| Oleanolic acid | FTO |
| Oleanolic acid | FUT7 |
| Oleanolic acid | FYN |
| Oleanolic acid | GABT |
| Oleanolic acid | GSTA1 |
| Oleanolic acid | HCAR3 |
| Oleanolic acid | HIF1N |
| Oleanolic acid | IBP1 |
| Oleanolic acid | IBP2 |
| Oleanolic acid | IBP4 |
| Oleanolic acid | IBP3 |
| Oleanolic acid | IBP5 |
| Oleanolic acid | IBP6 |
| Oleanolic acid | KCMA1 |
| Oleanolic acid | KDM2A |
| Oleanolic acid | KDM2B |
| Oleanolic acid | KDM3A |
| Oleanolic acid | KDM4A |
| Oleanolic acid | KDM4D |
| Oleanolic acid | KDM4E |
| Oleanolic acid | KDM5A |
| Oleanolic acid | KDM4C |
| Oleanolic acid | KDM5B |
| Oleanolic acid | KDM5C |
| Oleanolic acid | KDM6B |
| Oleanolic acid | KKCC2 |
| Oleanolic acid | KMO |
| Oleanolic acid | KS6B2 |
| Oleanolic acid | LOX15 |
| Oleanolic acid | MPIP2 |
| Oleanolic acid | NEUR3 |
| Oleanolic acid | NR1H4 |
| Oleanolic acid | NR4A1 |
| Oleanolic acid | NR4A2 |
| Oleanolic acid | NSD2 |
| Oleanolic acid | OXDD |
| Oleanolic acid | P4HA1 |
| Oleanolic acid | P4HTM |
| Oleanolic acid | PA24B |
| Oleanolic acid | PA2GD |
| Oleanolic acid | PAI1 |
| Oleanolic acid | PLAP |
| Oleanolic acid | PLEC |
| Oleanolic acid | POLH |
| Oleanolic acid | POLI |
| Oleanolic acid | PPBN |
| Oleanolic acid | PPBT |
| Oleanolic acid | RARA |
| Oleanolic acid | RARB |
| Oleanolic acid | RARG |
| Oleanolic acid | RHOA |
| Oleanolic acid | RXRA |
| Oleanolic acid | RXRB |
| Oleanolic acid | RXRG |
| Oleanolic acid | S22A6 |
| Oleanolic acid | S5A2 |
| Oleanolic acid | SSDH |
| Oleanolic acid | SYUA |
| Oleanolic acid | TOP1 |
| Oleanolic acid | TPMT |
| Oleanolic acid | TTHY |
| Oleanolic acid | CAH2 |
| Oleanolic acid | CAH7 |
| Oleanolic acid | CAH1 |
| Oleanolic acid | CAH6 |
| Oleanolic acid | CAH12 |
| Oleanolic acid | CAH14 |
| Oleanolic acid | CAH9 |
| Oleanolic acid | CAH2 |
| Oleanolic acid | CHLE |
| Oleanolic acid | GLCM |
| Oleanolic acid | CAH1 |
| Oleanolic acid | DDX6 |
| Oleanolic acid | PDE4B |
| Oleanolic acid | CDK2 |
| Oleanolic acid | GALK1 |
| Oleanolic acid | CFAB |
| Oleanolic acid | PH4H |
| Oleanolic acid | MAOM |
| Oleanolic acid | BACE1 |
| Oleanolic acid | AMY1 |
| Oleanolic acid | PNPH |
| Oleanolic acid | ANGI |
| Oleanolic acid | PIM1 |
| Oleanolic acid | PDE4D |
| Oleanolic acid | NOS3 |
| Oleanolic acid | BCAT2 |
| Oleanolic acid | CAH4 |
| Oleanolic acid | TY3H |
| Oleanolic acid | XCT |
| Oleanolic acid | XPF |
| Caffeic acid | A4 |
| Caffeic acid | ABCG2 |
| Caffeic acid | AK1BA |
| Caffeic acid | AK1C4 |
| Caffeic acid | AK1C3 |
| Caffeic acid | ALDR |
| Caffeic acid | AOFA |
| Caffeic acid | AOFB |
| Caffeic acid | BACE1 |
| Caffeic acid | CAH14 |
| Caffeic acid | CAH12 |
| Caffeic acid | CAH1 |
| Caffeic acid | CAH3 |
| Caffeic acid | CAH2 |
| Caffeic acid | CAH4 |
| Caffeic acid | CAH5B |
| Caffeic acid | CAH5A |
| Caffeic acid | CAH6 |
| Caffeic acid | CAH7 |
| Caffeic acid | CAH9 |
| Caffeic acid | CH60 |
| Caffeic acid | CISD1 |
| Caffeic acid | CP1B1 |
| Caffeic acid | CP26B |
| Caffeic acid | DAPK2 |
| Caffeic acid | DCOR |
| Caffeic acid | DHPR |
| Caffeic acid | DYN1 |
| Caffeic acid | EGFR |
| Caffeic acid | EP300 |
| Caffeic acid | ERCC1 |
| Caffeic acid | ESR1 |
| Caffeic acid | ESR2 |
| Caffeic acid | FOS |
| Caffeic acid | FUT7 |
| Caffeic acid | GBB1 |
| Caffeic acid | GBG2 |
| Caffeic acid | GBRR2 |
| Caffeic acid | GSTA1 |
| Caffeic acid | HCAR2 |
| Caffeic acid | HDAC2 |
| Caffeic acid | HS71A |
| Caffeic acid | IBP2 |
| Caffeic acid | IBP1 |
| Caffeic acid | IBP4 |
| Caffeic acid | IBP5 |
| Caffeic acid | IBP3 |
| Caffeic acid | IBP6 |
| Caffeic acid | JUN |
| Caffeic acid | KDM4E |
| Caffeic acid | KKCC2 |
| Caffeic acid | LGUL |
| Caffeic acid | LOX15 |
| Caffeic acid | MARK4 |
| Caffeic acid | MMP1 |
| Caffeic acid | MMP2 |
| Caffeic acid | MMP9 |
| Caffeic acid | NEMO |
| Caffeic acid | NF2L2 |
| Caffeic acid | NFKB1 |
| Caffeic acid | NOX4 |
| Caffeic acid | NQO2 |
| Caffeic acid | NR0B2 |
| Caffeic acid | NSD2 |
| Caffeic acid | PE2R2 |
| Caffeic acid | PE2R3 |
| Caffeic acid | PE2R4 |
| Caffeic acid | PLAP |
| Caffeic acid | PPBI |
| Caffeic acid | PPBN |
| Caffeic acid | PPBT |
| Caffeic acid | PSDE |
| Caffeic acid | PTN11 |
| Caffeic acid | PTN1 |
| Caffeic acid | Q9UM81 |
| Caffeic acid | RARA |
| Caffeic acid | RARB |
| Caffeic acid | RHOA |
| Caffeic acid | ST17B |
| Caffeic acid | SYUA |
| Caffeic acid | TAU |
| Caffeic acid | TBB1 |
| Caffeic acid | TBB3 |
| Caffeic acid | TF |
| Caffeic acid | TNR1A |
| Caffeic acid | TTHY |
| Caffeic acid | TY3H |
| Caffeic acid | CAH2 |
| Caffeic acid | LOX5 |
| Caffeic acid | CAH7 |
| Caffeic acid | CAH1 |
| Caffeic acid | CAH6 |
| Caffeic acid | MMP9 |
| Caffeic acid | CAH12 |
| Caffeic acid | MMP1 |
| Caffeic acid | MMP2 |
| Caffeic acid | PTN1 |
| Caffeic acid | CAH14 |
| Caffeic acid | CAH9 |
| Caffeic acid | CAH5B |
| Caffeic acid | SEPR |
| Caffeic acid | PIM1 |
| Caffeic acid | CMA1 |
| Caffeic acid | EGFR |
| Caffeic acid | AURKA |
| Caffeic acid | CDK2 |
| Caffeic acid | PDPK1 |
| Caffeic acid | NGAL |
| Caffeic acid | MTAP |
| Caffeic acid | PH4H |
| Caffeic acid | BACE1 |
| Caffeic acid | CATD |
| Caffeic acid | PTN1 |
| Caffeic acid | CHK1 |
| Caffeic acid | TYPH |
| Caffeic acid | BMP7 |
| Caffeic acid | ESR1 |
| Caffeic acid | CAH2 |
| Caffeic acid | MMP8 |
| Caffeic acid | CAH5A |
| Caffeic acid | XDH |
| Caffeic acid | XPF |
| Methylprotodioscin_qt | ANDR |
| Methylprotodioscin_qt | CBG |
| Methylprotodioscin_qt | CP17A |
| Methylprotodioscin_qt | DPOLA |
| Methylprotodioscin_qt | EBP |
| Methylprotodioscin_qt | ESR1 |
| Methylprotodioscin_qt | ESR2 |
| Methylprotodioscin_qt | G6PD |
| Methylprotodioscin_qt | IL2 |
| Methylprotodioscin_qt | MRP4 |
| Methylprotodioscin_qt | NPCL1 |
| Methylprotodioscin_qt | NR1H3 |
| Methylprotodioscin_qt | RORA |
| Methylprotodioscin_qt | S5A2 |
| Methylprotodioscin_qt | SHBG |
| Methylprotodioscin_qt | IL2 |
| Methylprotodioscin_qt | NR1H3 |
| Methylprotodioscin_qt | NR1H2 |
| Methylprotodioscin_qt | VDR |
| Methylprotodioscin_qt | DHI1 |
| Methylprotodioscin_qt | PTN1 |
| Methylprotodioscin_qt | ANDR |
| Methylprotodioscin_qt | VTDB |
| Methylprotodioscin_qt | CNR1 |
| Methylprotodioscin_qt | FAAH1 |
| Methylprotodioscin_qt | STAT3 |
| Methylprotodioscin_qt | CP17A |
| Methylprotodioscin_qt | ITAL |
| Methylprotodioscin_qt | PSN2 |
| Methylprotodioscin_qt | PEN2 |
| Methylprotodioscin_qt | NICA |
| Methylprotodioscin_qt | APH1A |
| Methylprotodioscin_qt | PSN1 |
| Methylprotodioscin_qt | APH1B |
| Methylprotodioscin_qt | CAH2 |
| Methylprotodioscin_qt | APOA2 |
| Methylprotodioscin_qt | ALBU |
| Methylprotodioscin_qt | AK1C2 |
| Methylprotodioscin_qt | BMP2 |
| Methylprotodioscin_qt | STS |
| Methylprotodioscin_qt | CASP7 |
| Methylprotodioscin_qt | AOFB |
| Methylprotodioscin_qt | EST1 |
| Methylprotodioscin_qt | HPGDS |
| Methylprotodioscin_qt | PIM1 |
| Methylprotodioscin_qt | PK3CG |
| Methylprotodioscin_qt | CFAB |
| Methylprotodioscin_qt | KIF11 |
| Methylprotodioscin_qt | GSTP1 |
| Methylprotodioscin_qt | MK10 |
| Methylprotodioscin_qt | ITAL |
| Methylprotodioscin_qt | CD5R1 |
| Methylprotodioscin_qt | VTDB |
| Methylprotodioscin_qt | ANDR |
| Methylprotodioscin_qt | THRB |
| Methylprotodioscin_qt | SHH |
| Methylprotodioscin_qt | SRBP2 |
| coumarin | AHR |
| coumarin | AOFA |
| coumarin | AOFB |
| coumarin | CAH13 |
| coumarin | CAH12 |
| coumarin | CAH14 |
| coumarin | CAH1 |
| coumarin | CAH3 |
| coumarin | CAH2 |
| coumarin | CAH4 |
| coumarin | CAH5A |
| coumarin | CAH5B |
| coumarin | CAH6 |
| coumarin | CAH7 |
| coumarin | CAH9 |
| coumarin | CP1B1 |
| coumarin | DRD4 |
| coumarin | ERAP1 |
| coumarin | HNF4A |
| coumarin | KCNA1 |
| coumarin | KCNA2 |
| coumarin | KCNA3 |
| coumarin | KCNA4 |
| coumarin | KCNA5 |
| coumarin | CAH2 |
| coumarin | CAH7 |
| coumarin | CAH1 |
| coumarin | CAH3 |
| coumarin | CAH6 |
| coumarin | CAH12 |
| coumarin | CAH14 |
| coumarin | CAH9 |
| coumarin | CAH4 |
| coumarin | CAH13 |
| coumarin | CAH5B |
| coumarin | PPIA |
| coumarin | CHLE |
| coumarin | BCAT2 |
| coumarin | PPARG |
| coumarin | MK14 |
| coumarin | THRB |
| coumarin | PTN1 |
| coumarin | MAP2 |
| coumarin | SRC |
| coumarin | ALDR |
| coumarin | TGM3 |
| coumarin | PCKGC |
| coumarin | ALBU |
| coumarin | PDE4D |
| coumarin | CP2C9 |
| coumarin | PDE4B |
| coumarin | FKB1A |
| coumarin | HS90A |
| coumarin | VGFR2 |
| coumarin | CAH5A |
| coumarin | KCNA7 |
| coumarin | KCNN1 |
| coumarin | KCNN2 |
| coumarin | MIF |
| coumarin | NFKB1 |
| coumarin | NQO1 |
| coumarin | PCSK7 |
| coumarin | PRGR |
| Kaempferide | 3MG |
| Kaempferide | A4 |
| Kaempferide | ABCG2 |
| Kaempferide | AK1BA |
| Kaempferide | ALDH2 |
| Kaempferide | ALDR |
| Kaempferide | AMY1 |
| Kaempferide | AOFA |
| Kaempferide | AOFB |
| Kaempferide | APEX1 |
| Kaempferide | ASAH1 |
| Kaempferide | BGLR |
| Kaempferide | CAH13 |
| Kaempferide | CAH12 |
| Kaempferide | CAH14 |
| Kaempferide | CAH3 |
| Kaempferide | CAH4 |
| Kaempferide | CAH5A |
| Kaempferide | CAH6 |
| Kaempferide | CAH7 |
| Kaempferide | CALM1 |
| Kaempferide | CBR1 |
| Kaempferide | CCR4 |
| Kaempferide | CISD1 |
| Kaempferide | CP19A |
| Kaempferide | CP1B1 |
| Kaempferide | CP2C8 |
| Kaempferide | CREB1 |
| Kaempferide | DAPK1 |
| Kaempferide | DHB2 |
| Kaempferide | ELAV1 |
| Kaempferide | ERAP1 |
| Kaempferide | ERN1 |
| Kaempferide | ERR1 |
| Kaempferide | ERR2 |
| Kaempferide | ESR1 |
| Kaempferide | ESR2 |
| Kaempferide | FACE2 |
| Kaempferide | GPR35 |
| Kaempferide | GRK6 |
| Kaempferide | HNF4A |
| Kaempferide | IL2 |
| Kaempferide | KCND3 |
| Kaempferide | KDM4E |
| Kaempferide | KLK7 |
| Kaempferide | LGUL |
| Kaempferide | LOX12 |
| Kaempferide | LOX15 |
| Kaempferide | LOX5 |
| Kaempferide | LRP6 |
| Kaempferide | MDHC |
| Kaempferide | MDR1 |
| Kaempferide | MOT4 |
| Kaempferide | MRP1 |
| Kaempferide | NEK6 |
| Kaempferide | NMUR2 |
| Kaempferide | NOX4 |
| Kaempferide | NQO1 |
| Kaempferide | NR4A2 |
| Kaempferide | NUAK1 |
| Kaempferide | PDIA1 |
| Kaempferide | PDK1 |
| Kaempferide | PDK3 |
| Kaempferide | PDK4 |
| Kaempferide | PERM |
| Kaempferide | PKN1 |
| Kaempferide | PLGF |
| Kaempferide | PON1 |
| Kaempferide | PPAC |
| Kaempferide | PPBI |
| Kaempferide | PTPRS |
| Kaempferide | S22AC |
| Kaempferide | SIAT1 |
| Kaempferide | T2R31 |
| Kaempferide | TAU |
| Kaempferide | TERT |
| Kaempferide | TNKS1 |
| Kaempferide | TOP2A |
| Kaempferide | TTHY |
| Kaempferide | MDR1 |
| Kaempferide | CP1B1 |
| Kaempferide | MRP1 |
| Kaempferide | CAH7 |
| Kaempferide | DHB2 |
| Kaempferide | CAH12 |
| Kaempferide | CAH4 |
| Kaempferide | XDH |
| Kaempferide | CAH2 |
| Kaempferide | ALDR |
| Kaempferide | MCL1 |
| Kaempferide | CP19A |
| Kaempferide | S22AC |
| Kaempferide | DHB1 |
| Kaempferide | TYRO |
| Kaempferide | HCK |
| Kaempferide | ESR1 |
| Kaempferide | CAH2 |
| Kaempferide | PIM1 |
| Kaempferide | CHLE |
| Kaempferide | CFAB |
| Kaempferide | THRB |
| Kaempferide | EGFR |
| Kaempferide | PPIA |
| Kaempferide | TTHY |
| Kaempferide | CDK6 |
| Kaempferide | CDK2 |
| Kaempferide | MK10 |
| Kaempferide | MK08 |
| Kaempferide | AOFB |
| Kaempferide | MMP3 |
| Kaempferide | GLCM |
| Kaempferide | CD5R1 |
| Kaempferide | CAH1 |
| Kaempferide | AURKA |
| Kaempferide | TYRO |
| Kaempferide | VEGFA |
| Kaempferide | XDH |
| Gramine | 5HT1A |
| Gramine | 5HT1B |
| Gramine | 5HT1D |
| Gramine | 5HT1F |
| Gramine | 5HT1E |
| Gramine | 5HT2A |
| Gramine | 5HT2B |
| Gramine | 5HT2C |
| Gramine | 5HT4R |
| Gramine | 5HT5A |
| Gramine | 5HT6R |
| Gramine | 5HT7R |
| Gramine | ACES |
| Gramine | ACE |
| Gramine | ADA12 |
| Gramine | AHR |
| Gramine | AKT1 |
| Gramine | AKT3 |
| Gramine | BRS3 |
| Gramine | CATB |
| Gramine | CATL1 |
| Gramine | CFTR |
| Gramine | CHLE |
| Gramine | CP2D6 |
| Gramine | DRD2 |
| Gramine | DRD3 |
| Gramine | DRD4 |
| Gramine | ECE1 |
| Gramine | EDNRA |
| Gramine | GASR |
| Gramine | GCR |
| Gramine | GHSR |
| Gramine | GPR84 |
| Gramine | HDA11 |
| Gramine | HDAC1 |
| Gramine | HDAC2 |
| Gramine | HDAC3 |
| Gramine | JUN |
| Gramine | KCC2D |
| Gramine | KGP1 |
| Gramine | KI20A |
| Gramine | KPCB |
| Gramine | KPCD |
| Gramine | KPCE |
| Gramine | KPCG |
| Gramine | KPCL |
| Gramine | KPCZ |
| Gramine | MC3R |
| Gramine | MC4R |
| Gramine | MC5R |
| Gramine | MCR |
| Gramine | MMP14 |
| Gramine | MMP15 |
| Gramine | MMP17 |
| Gramine | MMP1 |
| Gramine | MMP26 |
| Gramine | MMP2 |
| Gramine | MMP8 |
| Gramine | MMP9 |
| Gramine | MRCKA |
| Gramine | NCOR2 |
| Gramine | NEP |
| Gramine | NK1R |
| Gramine | NK2R |
| Gramine | NMBR |
| Gramine | NUAK1 |
| Gramine | OPRM |
| Gramine | PDIA1 |
| Gramine | PERM |
| Gramine | PPIB |
| Gramine | PSA |
| Gramine | PSB9 |
| Gramine | RAD51 |
| Gramine | RUVB1 |
| Gramine | SC6A2 |
| Gramine | SC6A3 |
| Gramine | SC6A4 |
| Gramine | SIA4A |
| Gramine | SPRE |
| Gramine | SSR1 |
| Gramine | SSR2 |
| Gramine | SSR3 |
| Gramine | SSR4 |
| Gramine | SSR5 |
| Gramine | 5HT2A |
| Gramine | 5HT2C |
| Gramine | 5HT2B |
| Gramine | DRD2 |
| Gramine | DRD3 |
| Gramine | ACHA4 |
| Gramine | ACHB2 |
| Gramine | AOFA |
| Gramine | AOFB |
| Gramine | PERM |
| Gramine | SC6A4 |
| Gramine | SC6A3 |
| Gramine | 5HT1A |
| Gramine | SGMR1 |
| Gramine | ACHA7 |
| Gramine | 5HT6R |
| Gramine | TNR1A |
| Dihydrokaempferide | 5HT1A |
| Dihydrokaempferide | 5HT1B |
| Dihydrokaempferide | 5HT1D |
| Dihydrokaempferide | 5HT1F |
| Dihydrokaempferide | 5HT1E |
| Dihydrokaempferide | 5HT2B |
| Dihydrokaempferide | 5HT2A |
| Dihydrokaempferide | 5HT2C |
| Dihydrokaempferide | 5HT4R |
| Dihydrokaempferide | 5HT5A |
| Dihydrokaempferide | 5HT6R |
| Dihydrokaempferide | 5HT7R |
| Dihydrokaempferide | ACES |
| Dihydrokaempferide | ACE |
| Dihydrokaempferide | ADA12 |
| Dihydrokaempferide | AHR |
| Dihydrokaempferide | AKT1 |
| Dihydrokaempferide | AKT3 |
| Dihydrokaempferide | BRS3 |
| Dihydrokaempferide | CATB |
| Dihydrokaempferide | CATL1 |
| Dihydrokaempferide | CFTR |
| Dihydrokaempferide | CHLE |
| Dihydrokaempferide | CP2D6 |
| Dihydrokaempferide | DRD2 |
| Dihydrokaempferide | DRD3 |
| Dihydrokaempferide | DRD4 |
| Dihydrokaempferide | ECE1 |
| Dihydrokaempferide | EDNRA |
| Dihydrokaempferide | GASR |
| Dihydrokaempferide | GCR |
| Dihydrokaempferide | GHSR |
| Dihydrokaempferide | GPR84 |
| Dihydrokaempferide | HDA11 |
| Dihydrokaempferide | HDAC1 |
| Dihydrokaempferide | HDAC2 |
| Dihydrokaempferide | HDAC3 |
| Dihydrokaempferide | JUN |
| Dihydrokaempferide | KCC2D |
| Dihydrokaempferide | KGP1 |
| Dihydrokaempferide | KI20A |
| Dihydrokaempferide | KPCB |
| Dihydrokaempferide | KPCD |
| Dihydrokaempferide | KPCE |
| Dihydrokaempferide | KPCG |
| Dihydrokaempferide | KPCL |
| Dihydrokaempferide | KPCZ |
| Dihydrokaempferide | MC3R |
| Dihydrokaempferide | MC4R |
| Dihydrokaempferide | MC5R |
| Dihydrokaempferide | MCR |
| Dihydrokaempferide | MMP14 |
| Dihydrokaempferide | MMP15 |
| Dihydrokaempferide | MMP17 |
| Dihydrokaempferide | MMP1 |
| Dihydrokaempferide | MMP26 |
| Dihydrokaempferide | MMP2 |
| Dihydrokaempferide | MMP8 |
| Dihydrokaempferide | MMP9 |
| Dihydrokaempferide | MRCKA |
| Dihydrokaempferide | NCOR2 |
| Dihydrokaempferide | NEP |
| Dihydrokaempferide | NK1R |
| Dihydrokaempferide | NK2R |
| Dihydrokaempferide | NMBR |
| Dihydrokaempferide | NUAK1 |
| Dihydrokaempferide | OPRM |
| Dihydrokaempferide | PDIA1 |
| Dihydrokaempferide | PERM |
| Dihydrokaempferide | PPIB |
| Dihydrokaempferide | PSA |
| Dihydrokaempferide | PSB9 |
| Dihydrokaempferide | RAD51 |
| Dihydrokaempferide | RUVB1 |
| Dihydrokaempferide | SC6A2 |
| Dihydrokaempferide | SC6A3 |
| Dihydrokaempferide | SC6A4 |
| Dihydrokaempferide | SIA4A |
| Dihydrokaempferide | SPRE |
| Dihydrokaempferide | SSR1 |
| Dihydrokaempferide | SSR2 |
| Dihydrokaempferide | SSR3 |
| Dihydrokaempferide | SSR4 |
| Dihydrokaempferide | SSR5 |
| Dihydrokaempferide | PDE5A |
| Dihydrokaempferide | SNRPA |
| Dihydrokaempferide | PTN1 |
| Dihydrokaempferide | CDK2 |
| Dihydrokaempferide | PDE4B |
| Dihydrokaempferide | TYSY |
| Dihydrokaempferide | SRC |
| Dihydrokaempferide | FOLH1 |
| Dihydrokaempferide | CAH2 |
| Dihydrokaempferide | GLRX1 |
| Dihydrokaempferide | PDE4D |
| Dihydrokaempferide | CDK6 |
| Dihydrokaempferide | TTHY |
| Dihydrokaempferide | CBR1 |
| Dihydrokaempferide | CATK |
| Dihydrokaempferide | LDHB |
| Dihydrokaempferide | ALBU |
| Dihydrokaempferide | SODM |
| Dihydrokaempferide | UMPS |
| Dihydrokaempferide | PDPK1 |
| Dihydrokaempferide | TNR1A |
| Diosgenin | ANDR |
| Diosgenin | CBG |
| Diosgenin | CP17A |
| Diosgenin | CP19A |
| Diosgenin | DPOLA |
| Diosgenin | EBP |
| Diosgenin | ESR1 |
| Diosgenin | ESR2 |
| Diosgenin | G6PD |
| Diosgenin | IF4A1 |
| Diosgenin | IL2 |
| Diosgenin | MRP4 |
| Diosgenin | NPCL1 |
| Diosgenin | NR1H3 |
| Diosgenin | RORA |
| Diosgenin | S5A2 |
| Diosgenin | SHBG |
| Diosgenin | SHH |
| Diosgenin | APOA2 |
| Diosgenin | STS |
| Diosgenin | MAPK2 |
| Diosgenin | ALBU |
| Diosgenin | AK1C2 |
| Diosgenin | BMP2 |
| Diosgenin | PIM1 |
| Diosgenin | AOFB |
| Diosgenin | ANDR |
| Diosgenin | CAH2 |
| Diosgenin | THB |
| Diosgenin | KIF11 |
| Diosgenin | ITAL |
| Diosgenin | EST1 |
| Diosgenin | CASP7 |
| Diosgenin | PTN1 |
| Diosgenin | MK01 |
| Diosgenin | CCNA2 |
| Diosgenin | CP19A |
| Diosgenin | SRBP2 |
| Diosgenin | NR1H3 |
| Diosgenin | IL2 |
| Diosgenin | SHH |
| Diosgenin | CP51A |
| Diosgenin | RORG |
| Diosgenin | RORA |
| Diosgenin | STAT3 |
| Diosgenin | HMDH |
| Diosgenin | PTN1 |
| Diosgenin | CNR1 |
| Diosgenin | ALK |
| Diosgenin | DHI1 |
| Diosgenin | VDR |
| Diosgenin | MDM2 |
| Diosgenin | ERG7 |

**Supplementary Table 4** 86 eighty-six PID-related targets from Drugbank, GeneCards and DisGeNET.

| Common name | Uniprot ID |
| --- | --- |
| ABCB1 | P08183 |
| CYP3A4 | P08684 |
| ABCC2 | Q92887 |
| SLC22A6 | Q4U2R8 |
| ALB | P02768 |
| MPO | P05164 |
| PTGS2 | P35354 |
| STAT3 | P40763 |
| MMP9 | P14780 |
| PGR | P06401 |
| TNF | P01375 |
| IL6 | P05231 |
| IL2 | P60568 |
| PLAUR | Q03405 |
| MMP2 | P08253 |
| LCN2 | P80188 |
| CTSB | P07858 |
| PADI4 | Q9UM07 |
| pbpC | P42971 |
| SLC15A1 | P46059 |
| SLC15A2 | Q16348 |
| SLC22A8 | Q8TCC7 |
| SLC22A11 | Q9NSA0 |
| SLC22A7 | Q9Y694 |
| pbp1b | Q7CRA4 |
| pbp2a | Q8DNB6 |
| pbpA | Q8DR59 |
| penA | P0A3M6 |
|  | B2ZTR6 |
| pbp3 | Q75Y35 |
| mrcA | P02918 |
| mrcB | P02919 |
| ftsI | P0AD68 |
| dacA | P0AEB2 |
| dacB | P24228 |
| dacC | P08506 |
| pbpG | P0AFI5 |
| penP | P00808 |
| bla | P62594 |
| bla | P0AD63 |
| IL4 | P05112 |
| TLR4 | O00206 |
| TIRAP | P58753 |
| MYD88 | Q99836 |
| CXCL12 | P48061 |
| ROM1 | Q03395 |
| PTX3 | P26022 |
| TLR1 | Q15399 |
| TLR2 | O60603 |
| SCGB1A1 | P11684 |
| WAS | P42768 |
| P2RY14 | Q15391 |
| TLR6 | Q9Y2C9 |
| NOD1 | Q9Y239 |
| MAN1B1 | Q9UKM7 |
| HSPA14 | Q0VDF9 |
| CTSA | P10619 |
| PIK3CD | O00329 |
| CD247 | P20963 |
| CD19 | P15391 |
| CD40LG | P29965 |
| CHI3L1 | P36222 |
| CRP | P02741 |
| CST3 | P01034 |
| HSPA4 | P34932 |
| HSPD1 | P10809 |
| IFNG | P01579 |
| IL1A | P01583 |
| IL2RG | P31785 |
| TGFB1 | P01137 |
| IL1B | P01584 |
| IL10 | P22301 |
| CCL2 | P13500 |
| CXCL8 | P10145 |
| ELN | P15502 |
| IL13 | P35225 |
| CLEC3B | P05452 |
| CDH1 | P12830 |
| HLA-DQB1 | P01920 |
| STAT1 | P42224 |
| ERBB2 | P04626 |
| MAN1B1 | Q9UKM7 |
| TF | P02787 |
| LACTB | P83111 |
| AQP1 | P29972 |
| MAPK1 | P28482 |

**Supplementary Table 5** Glide Gscore of active compounds docking with PID targets.

| Title | Compounds | LCN2  (PDB ID: 1x89) | PTGS2  (PDB ID: 5kir) | MAPK1  (PDB ID: 5ax3) | TNF  (PDB ID: 2az5) | MMP-9  (PDB ID: 5ue4) |
| --- | --- | --- | --- | --- | --- | --- |
|  | Contrast | -7.190 | -9.800 | -8.713 | -7.879 | -8.661 |
| **1** | Engeletin | -6.218 | 0 | ***-8.342*** | -6.822 | -4.672 |
| **2** | Isoengeletin | -4.969 | -7.716 | ***-7.195*** | -6.56 | -5.218 |
| 3 | Astilbin | -4.625 | 0 | -7.839 | -6.311 | -4.961 |
| **4** | Polydatin | -5.628 | -7.097 | ***-8.846*** | -7.398 | -8.988 |
| **5** | Resveratrol | -5.722 | -7.539 | -8.195 | -7.052 | -8.695 |
| 8 | Sarsasapogenin | -4.019 | 0 | -3.534 | -4.884 | -3.67 |
| **10** | Rutin | **-7.320** | 0 | -7.896 | **-7.743** | -5.962 |
| **11** | Kaempferol | -6.353 | -8.986 | -7.143 | -6.54 | ***-7.734*** |
| **12** | Dihydrokaempferol | -4.463 | -8.748 | ***-8.167*** | -7.37 | -6.428 |
| **17** | Quercetin | -6.353 | -7.707 | ***-8.367*** | -6.964 | -7.888 |
| **18** | Isorhamnetin | ***-6.347*** | ***-9.757*** | -7.995 | -6.679 | -7.644 |
| **21** | Piceatannol | -4.855 | -6.850 | -8.673 | -5.852 | -8.038 |
| **30** | Oxyresveratrol | -5.782 | -8.382 | -8.085 | -7.076 | **-9.241** |
| 38 | Butyl β-D-fructopyranoside | -4.714 | -5.321 | -5.094 | -4.887 | -4.992 |
| 39 | Syringic acid | -4.879 | -6.337 | -6.472 | -5.314 | -6.392 |
| 40 | Protocatechuic acid | -4.529 | -6.449 | -5.586 | -5.53 | -6.018 |
| 41 | Vanillic acid | -4.666 | -6.236 | -6.145 | -5.339 | -6.139 |
| 42 | 3,5-Dimethoxy-4-β-D-glucopyranosylcinnamic acid | -4.530 | -4.014 | -3.835 | -6.869 | -6.554 |
| **43** | Maackoline | -4.833 | 0 | -6.179 | -6.294 | -3.502 |
| **44** | Moracin M | -4.260 | -8.833 | ***-9.326*** | -6.654 | -7.923 |
| 45 | Gallic acid | -4.566 | -6.852 | -5.929 | -5.275 | -6.112 |
| 46 | Trans-2-hydroxycinnamic acid | -4.118 | -6.291 | -6.525 | -5.624 | -6.473 |
| 47 | Gentisic acid | -5.825 | -6.238 | -6.741 | -5.139 | -6.199 |
| **48** | Dihydroquercetin | -4.637 | -8.538 | -8.469 | -7.020 | -6.411 |
| 50 | Oleanolic acid | -4.494 | -6.444 | -5.810 | -5.271 | -6.018 |
| 51 | Caffeic acid | -4.685 | -6.351 | -5.659 | -5.727 | -6.541 |
| 53 | Methylprotodioscin_qt | -4.007 | 0 | -6.194 | -5.638 | -6.196 |
| 55 | Coumarin | -5.246 | -7.049 | -5.716 | -5.795 | -4.762 |
| 58 | Kaempferide | -4.703 | -7.801 | -7.133 | -6.444 | -7.962 |
| 60 | Gramine | -4.507 | -7.955 | -6.100 | -7.452 | -6.629 |
| 62 | Dihydrokaempferide | -4.744 | -7.638 | -6.505 | -6.312 | -6.282 |
| 63 | Diosgenin | -3.875 | 0 | -4.983 | -6.111 | -5.518 |
